# Electrically Conductive and Highly Stretchable Piezoresistive Polymer Nanocomposites via Oxidative Chemical Vapor Deposition

**DOI:** 10.1021/acsami.3c06015

**Published:** 2023-06-22

**Authors:** Adrivit Mukherjee, Afshin Dianatdar, Magdalena Z. Gładysz, Hamoon Hemmatpour, Mart Hendriksen, Petra Rudolf, Małgorzata K. Włodarczyk-Biegun, Marleen Kamperman, Ajay Giri Prakash Kottapalli, Ranjita K. Bose

**Affiliations:** † Chemical Product Engineering, Engineering and Technology Institute Groningen (ENTEG), University of Groningen, Nijenborgh 4, Groningen 9747 AG, The Netherlands; ‡ Polymer Science, Zernike Institute for Advanced Materials (ZIAM), University of Groningen, Nijenborgh 4, Groningen 9747 AG, The Netherlands; § Surfaces and Thin Films, Zernike Institute for Advanced Materials (ZIAM), University of Groningen, Nijenborgh 4, Groningen 9747 AG, The Netherlands; ∥ Advanced Production Engineering, Engineering and Technology Institute Groningen (ENTEG), University of Groningen, Nijenborgh 4, Groningen 9747 AG, The Netherlands

**Keywords:** oxidative chemical vapor deposition, polypyrrole, electrically conductive nanocomposite, electrospinning, interface, stretchable piezoresistive strain sensor, biocompatible

## Abstract

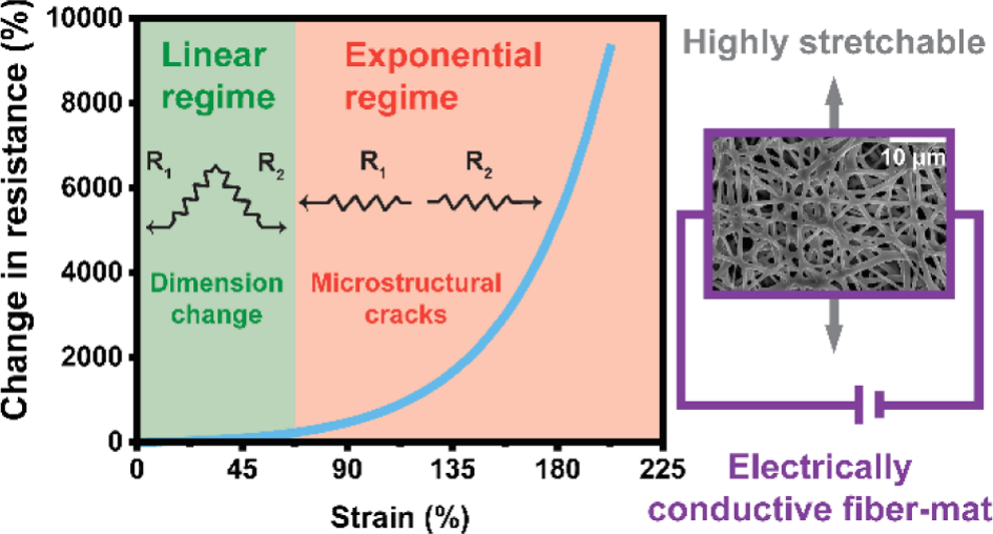

Electrically conductive polymer nanocomposites have been the subject of intense research due to their promising potential as piezoresistive biomedical sensors, leveraging their flexibility and biocompatibility. Although intrinsically conductive polymers such as polypyrrole (PPy) and polyaniline have emerged as lucrative candidates, they are extremely limited in their processability by conventional solution-based approaches. In this work, ultrathin nanostructured coatings of doped PPy are realized on polyurethane films of different architectures via oxidative chemical vapor deposition to develop stretchable and flexible resistance-based strain sensors. Holding the substrates perpendicular to the reactant flows facilitates diffusive transport and ensures excellent conformality of the interfacial integrated PPy coatings throughout the 3D porous electrospun fiber mats in a single step. This allows the mechanically robust (stretchability > 400%, with fatigue resistance up to 1000 cycles) nanocomposites to elicit a reversible change of electrical resistance when subjected to consecutive cycles of stretching and releasing. The repeatable performance of the strain sensor is linear due to dimensional changes of the conductive network in the low-strain regime (ε ≤ 50%), while the evolution of nano-cracks leads to an exponential increase, which is observed in the high-strain regime, recording a gauge factor as high as 46 at 202% elongational strain. The stretchable conductive polymer nanocomposites also show biocompatibility toward human dermal fibroblasts, thus providing a promising path for use as piezoresistive strain sensors and finding applications in biomedical applications such as wearable, skin-mountable flexible electronics.

## 1 Introduction

Flexible organic electronics play a crucial role in the fabrication of next-generation devices for a plethora of biomedical applications, such as tactile sensing,^1,2^ personalized healthcare monitoring,^3,4^ human–machine interactions,^5^ soft robotics,^6^ and electromagnetic devices with unique properties.^7−10^ This is due to their compatibility with the human body and sustained monitoring capabilities. Wearable and skin-conformable sensors that can withstand dynamic forces can be integrated with the human body and used to collect vital information about the physical, chemical, and biological signals with high precision and sensitivity.^11^ However, functional epidermal skin sensors must be highly sensitive, accommodate large strains (>100%), and have high mechanical compliance.^12^ Piezoresistive strain sensors using polymers have attracted much research attention as they offer a high degree of flexibility, stretchability, and relatively facile measurements suitable for practical implementations.^13−15^ Several approaches have been proposed to fabricate wearable piezoresistive strain sensors by integrating conductive nanomaterials into flexible polymer substrates.^16,17^ In these materials, the insulating polymers provide flexibility and mechanical compliance, whereas the conductive nanostructured materials provide the necessary electrical conductivity.^18−20^ There has been significant focus on the utilization of thermoplastic polyurethane (PU) as a flexible elastomeric support for diverse applications attributed to its high elasticity, strength, and durability, and hence it was chosen for this work.^21−23^ Additionally, good adhesion between the insulating polymer substrate and the conductive reinforcement is promoted as it facilitates improved load transfer across the interface to the active layer and prevents failure and delamination at the interface.^24−26^ Electrically conductive polymer nanocomposites developed by incorporating inorganic conductive reinforcements suffer from their inherent rigidity. Especially, these conductive fillers, such as silver nanoparticles, and carbon-based fillers, such as graphene and carbon nanotubes, exhibit poor solution processibility due to substrate incompatibility and agglomeration effects. This resulted in a tremendous shift in the recent research focus toward inherently conductive polymers (ICPs) such as polypyrrole (PPy), polyaniline (PANI), and poly(3,4-ethylene dioxythiophene) (PEDOT).^27−29^ PPy-based polymer composites have gained particular attention in the biomedical field due to several key benefits. One of the most significant advantages is the relatively stable, high electrical conductivity of PPy, depending upon the dopant used for synthesis. Additionally, the electrical properties of PPy can be easily tailored by adjusting the size and type of dopants used during the synthesis process, making it a highly versatile choice for various applications.^30^ Furthermore, PPy can be synthesized to be biocompatible, making it a popular choice for several biomedical applications. However, solution processing of these conductive polymers into well-defined thin films is challenging as they are brittle and insoluble in most common solvents in their unmodified form due to their conjugated backbone.^31^ Commonly applied solution-based techniques usually lead to inhomogeneity and dewetting of the final films due to surface tension effects. Moreover, the products are especially non-advantageous for biomedical applications due to residual toxic solvents. To overcome such limitations, vapor phase deposition technologies such as oxidative chemical vapor deposition (oCVD) are attractive synthesis and processing techniques for ICPs.^32,33^ The oCVD method is a state-of-the-art solvent-free deposition technique for ICPs, enabling a one-step synthesis and film formation of these polymers in their doped form.^32^ Recently, our group reported the synthesis and deposition of PPy using oCVD.^34^ The thin conductive films of PPy exhibited electrical conductivities as high as 180 S cm^–1^, while the final electrical conductivities, doping levels, and film morphologies could be tuned by altering the deposition parameters. Operated under mild reaction conditions, vapor phase deposition techniques such as oCVD allow for the diffusion of chemical reactants into complex micro and nanostructured geometries and can coat the inside surfaces of delicate porous substrates with a conductive coating.^32,35^ Numerous researchers have used this strategy to fabricate oCVD PEDOT and PANI-coated polymer nanocomposites for various applications. However, oCVD has rarely been employed to develop piezoresistive polymer nanocomposites with good mechanoelectrical properties that can withstand significant mechanical strains. In addition, existing literature shows a distinct lack of studies investigating the biocompatibility of conductive polymer coatings developed by the solvent-free oCVD process, specifically for their potential utilization in developing biomaterials. To the authors’ knowledge, only one report by Muralter et al. investigated the mechanoelectrical properties of oCVD PEDOT-coated polystyrene nanospheres.^36^ The sophisticated strain sensor design exhibited excellent sensitivities and moderate electrical conductivities but was limited to accommodating small bending strains (≈1%), and repetitive strain sensing was not reported.

In this work, we aim to fabricate free-standing, electrically conductive, stretchable, and biocompatible piezoresistive polymer nanocomposites that can withstand large strains. Herein, highly elastic and stretchable PU solid films and porous electrospun fiber mats with large surface areas constituting nanometer-scale individual fibers have been developed. These flexible and robust free-standing films with a thickness in the micrometer range were utilized as substrates for the oCVD of PPy. The deposition conditions were optimized to realize ultrathin conductive coatings of various thicknesses throughout the microstructure of the samples. The morphology of the PPy-coated electrospun fiber mats was imaged using scanning electron microscopy (SEM). The chemical composition of the resulting PPy has been explored by Fourier transform infrared (FTIR) spectroscopic analysis. Furthermore, the effects of the PPy coatings on the chemical composition and the thermal properties of the resulting polymer nanocomposites were investigated by attenuated total reflection (ATR) spectroscopy, X-ray photoelectron spectroscopy (XPS), thermogravimetric analysis (TGA), and differential scanning calorimetry (DSC). The later sections provide a comprehensive analysis of the mechanical and mechanoelectrical properties of the fabricated oCVD PPy-coated PU bulk and electrospun fiber mats while exploring the underlying mechanisms of piezoresistivity for the fabrication of strain sensors. Additionally, we have investigated the biocompatibility of the oCVD PPy coatings toward human dermal fibroblasts (HDFs) depending on the coating thickness to determine their potential for use as biomaterials.

## 2 Results and Discussion

### 2.1 Morphology of oCVD PPy-Coated Electrospun PU Fiber Mats

**Figure 1:**
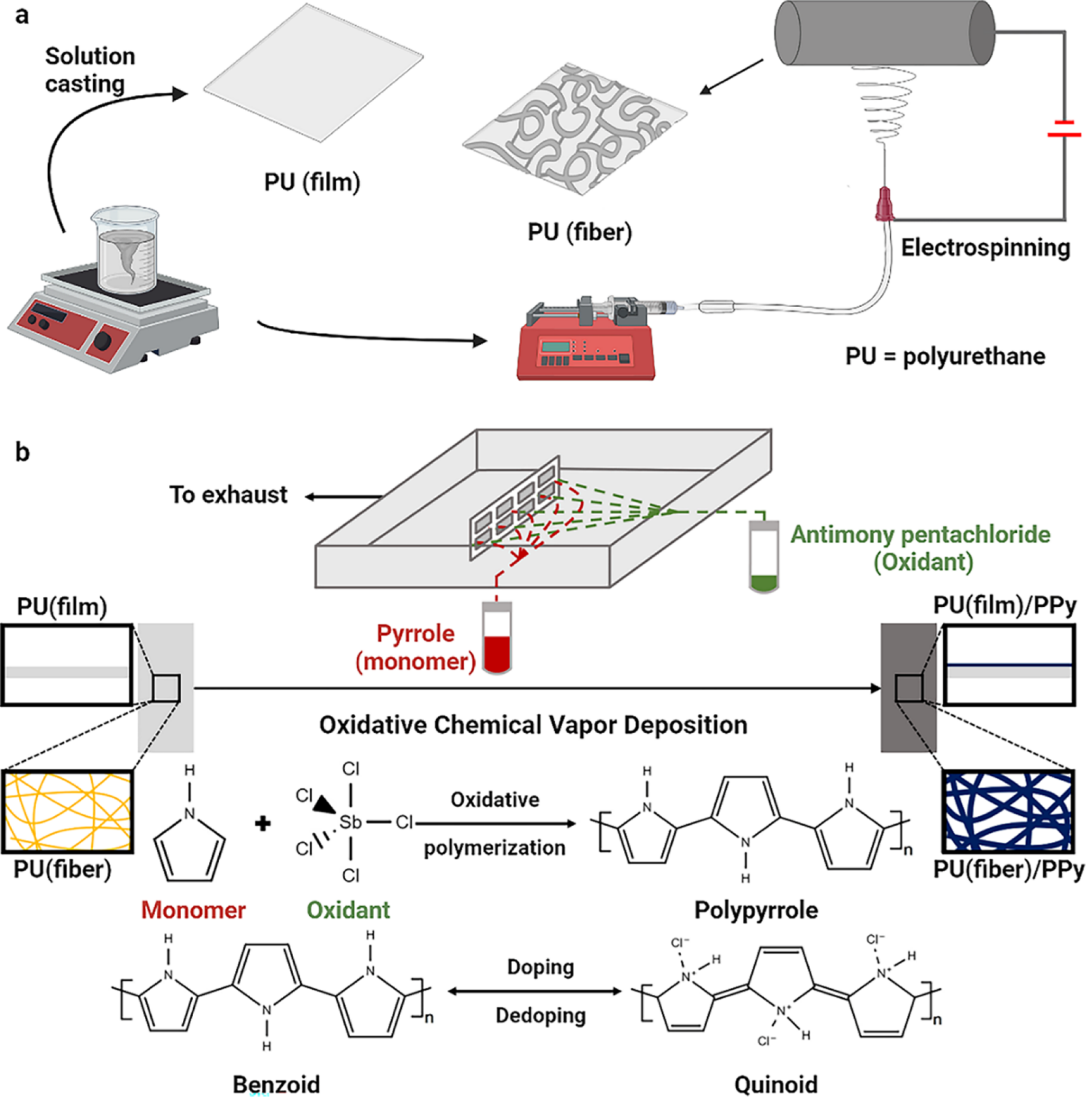
Two-step fabrication scheme of electrically conductive PU—(a) Developing PU solid films (film) by solvent casting and porous electrospun fiber mats (fiber); (b) oCVD of doped PPy using pyrrole and antimony pentachloride.

**Figure 2:**
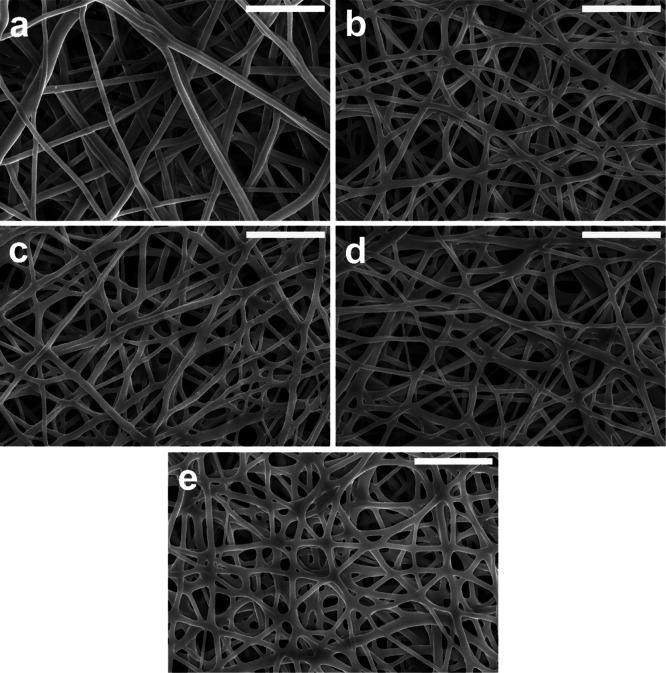
SEM images of the (a) uncoated electrospun PU fiber mat and the oCVD PPy-coated fiber mats for (b) 2.5; (c) 5; (d) 10; and (e) 20 min. The scale bar for all SEM images is 10 μm.

In the first step, free-standing PU substrates were developed as fiber mats using electrospinning, as shown in Figure 1a. A layer of PPy was then deposited on the flexible PU samples via oCVD (see Figure 1b) according to the specifications in Table 1. Figure 2a–e illustrate the SEM images of the microstructure of the pristine and the oCVD PPy-coated electrospun PU fiber mats. Figure 2a shows that the microstructure of the pristine electrospun PU fiber mat is characterized by randomly oriented fibers with a smooth surface and a high porosity due to the large inter-fiber distances. Figure 2b–e show the morphology of the oCVD PPy-coated electrospun fiber mats deposited for different time periods. SEM images of the oCVD PPy-coated electrospun PU fiber mats, obtained without additional conductive coatings, showed no apparent effects of charging, indicating that the samples had appreciable electrical conductivity (this will be discussed in detail in the later sections). The conformal coatings of PPy preserved the inter-fiber distance and, consequently, the porous microstructure of the electrospun fiber mats. Moreover, it can be observed that the fibers in the middle of the mat were also coated. This suggests that holding the samples perpendicular to the reactant vapor flows and using a high patch flow of nitrogen as a carrier gas were beneficial. This allowed the reactant vapors to diffuse throughout the entire porous microstructure and conformally coat the individual fibers. Furthermore, the oCVD PPy-coated fibers seem more flattened but exhibit a similarly smooth surface as the pristine fibers in the electrospun mat. These observations reveal a degree of conformality in PPy deposited by oCVD.^37^ This is in line with similar reports of conformally coated PANI into CNF fiber mats using oCVD.^38^ It can be observed from the SEM images that the individual fibers in the oCVD PPy-coated fiber mats are visibly thicker with increasing deposition time compared to the microstructure of the uncoated fiber mat. Figure S1a shows that the mean fiber diameter (averaged over 100 fibers) of the oCVD PPy-coated electrospun mats increases consistently with longer deposition times. Moreover, the variation among the fiber diameters before and after the coating process remains unchanged, indicating the homogeneity of the oCVD PPy depositions. A significant increase in the thickness of the fiber diameter from 640 ± 83 to 740 ± 104 nm was obtained and validated by a *t*-test with a 95% confidence interval (Figure S1a). The overall thickness of the conductive oCVD PPy coatings on the electrospun PU fiber mats was below 10 nm up to approximately 100 nm, depending on the duration of the experiment. The thickness of the coatings was plotted against the deposition time in Figure S1b, revealing an average deposition rate of 5 nm min^–1^.

**Table 1:** Summary of Sample Preparation Conditions via oCVD

sample	*F*_M_/*F*_O_	*F*_N_2__ (sccm)	pressure (mTorr)	temperature (°C)	time (min)
PU(film)/PPy	0.2	2	300	40	30
PU(fiber)/PPy(2.5)	0.2	30	300	40	2.5
PU(fiber)/PPy(5)	0.2	30	300	40	5
PU(fiber)/PPy(10)	0.2	30	300	40	10
PU(fiber)/PPy(20)	0.2	30	300	40	20

### 2.2 FTIR-ATR Spectroscopic Analysis of oCVD PPy-Coated PU Films

**Figure 3:**
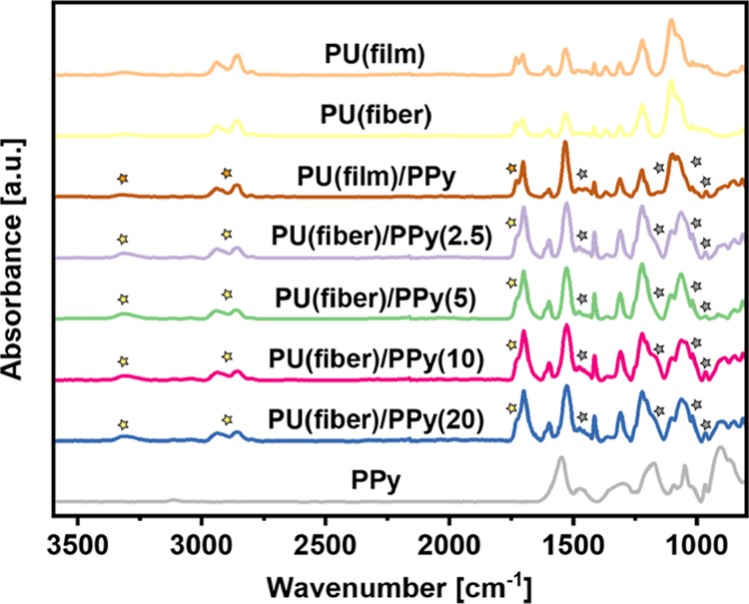
Baseline-corrected and normalized FTIR-ATR spectra of PU solid films (film) and fiber mats (fiber) coated with PPy via oCVD. The ATR spectrum of pristine PU (film, fiber) and the FTIR spectrum of pure PPy have been used for comparison.

Ultrathin coatings of PPy were deposited on the PU films and electrospun fiber mats via oCVD. Figure 3 shows the ATR spectra of the oCVD PPy-coated PU samples prepared according to the specifications listed in Table 1. The ATR spectra of the uncoated PU substrates and the FTIR spectra of pure PPy are used for comparison. The ATR spectra of both pristine PU samples are similar and illustrate the characteristic PU peaks (Table 2).^39,40^ The results suggest that despite using a strong oxidant, the oCVD process did not result in any observable degradation of the PU substrates.

**Table 2:** FTIR-ATR Peak Assignments for oCVD PPy-Coated PU Nanocomposites

wavenumbers (cm^–1^)	peak assignments	
	contributions from PU	contributions from oCVD PPy
3314	–NH stretching (H-bonded)	X
3110	X	–CH stretching
2940	–CH (symmetric) stretching	X
2855	–CH (anti-symmetric) stretching	X
1730	–C=O (free) stretching	X
1703	–C=O (H-bonded) stretching	X
1530	–NH (free) stretching	–C=C/C–C stretching
1455	–CH_2_ bonds	–CN stretching
1413, 1369	–CH_2_ bonds	X
1305	–CN deformation	–CH bending
1221	ether groups	X
1168	X	Pyrrole ring breathing
1095	X	–CH in-plane vibration
1105	ether groups	X
1019	X	deformation in the vibrational plane of −CH groups
966	X	pyrrole ring deformation

The FTIR absorbance spectrum of oCVD PPy is shown in Figure S2. The low-frequency region of the raw oCVD PPy (<1650 cm^–1^) exhibits sharp characteristic peaks, while the high-frequency part is devoid of any distinct peaks and is characterized by a sharp, monotonic rise in absorbance. The peak assignments of oCVD PPy from literature are more complex. Notably, while significant variations in the FTIR spectra for pristine PPy have been reported to be dependent on the synthesis methods, the spectra of PPy in its doped state bear remarkably close resemblances.^41−43^ In an early report, it was suggested that such a rise in absorbance is caused by the high excitation energies of the free charge carriers shrouding other contributions in this region and is a critical feature of doped PPy as compared to its pristine form.^44^ For further peak assignments of the oCVD PPy spectra (Table 2), the baseline-corrected and normalized absorbance spectra shown in Figure 3 are used.^34,43,45^ Despite the partial overlap in the IR spectra of PU and PPy, most peaks are evident, confirming the successful polymerization, doping, and deposition of oCVD PPy on both the PU film and fiber mat substrates.

**Figure 4:**
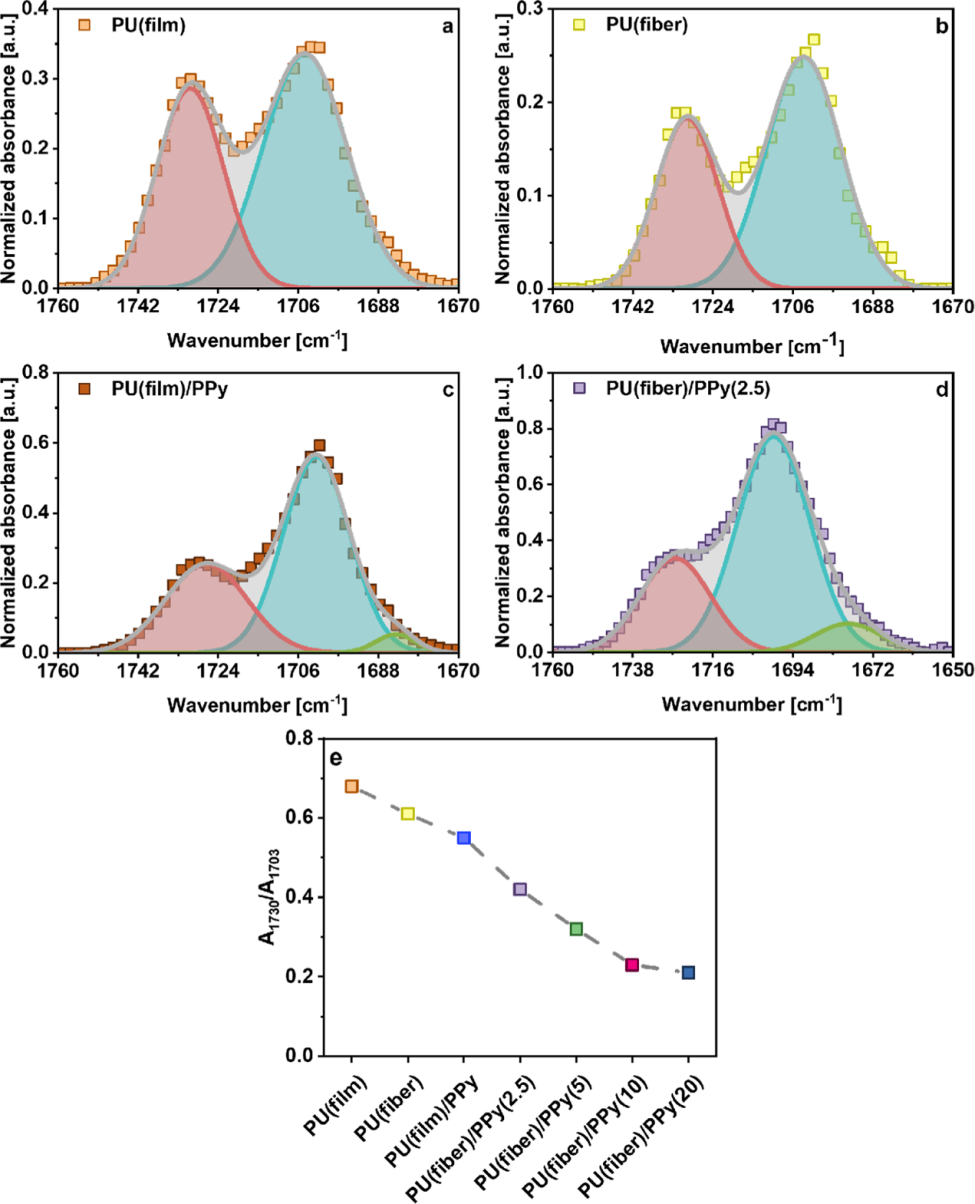
Peak deconvolutions of the free and hydrogen-bonded carbonyl peaks in the ATR spectra of (a) uncoated PU solid film (film); (b) uncoated PU electrospun fiber mat (fiber); (c) one side oCVD PPy-coated solid PU film (film); (d) PU electrospun fiber mat (fiber) coated with oCVD PPy throughout; and (e) the ratio of peak areas of the two carbonyl peaks (free carbonyl bonds at 1730 cm^–1^ and hydrogen-bonded carbonyl bonds at 1703 cm^–1^).

However, some differences exist between the spectra of the oCVD PPy-coated PU samples and their pristine counterparts. The broad peak centered around 3325 cm^–1^, attributed to the stretching of the −NH bonded groups in the spectra of the pristine PU samples, red-shifted to 3314 cm^–1^ after the coating process, suggesting a decrease in the average bond strength.^46^ Moreover, a significant alteration in the shapes of the carbonyl peaks originating from the underlying PU substrates (film, fiber) after the inclusion of oCVD PPy is evident in Figure 3. Figures 4a–d and S3b–d depict the peak deconvolutions of the carbonyl peaks in both the pristine and the oCVD PPy-coated PU substrates (film, fiber). The deconvoluted spectra reveal two Gaussian peaks centered around 1730 and 1703 cm^–1^, revealing the free and the hydrogen-bonded carbonyl peaks, respectively.^39^ In the oCVD PPy-coated PU (film, fiber), the hydrogen-bonded carbonyl peaks are significantly pronounced compared to the free carbonyl stretching peaks (Figure 4c,d). This suggests interactions between the −NH bonds of oCVD PPy coating and the −C=O groups from underlying PU substrates (film, fiber) via hydrogen bonding.^47^ This is also reflected in the ratio of the corresponding peak areas of the free and hydrogen-bonded carbonyl peaks (*A*_1730_/*A*_1703_) in the uncoated and-coated samples (Figure 4e). This is further corroborated by the emergence of a new peak around 1685 cm^–1^ in the ATR spectra of all the oCVD PPy-coated samples. This new peak is assigned to the hydrogen bonding between the −NH and −C=O groups.^48,49^ It can be observed that the oCVD PPy-coated electrospun PU fiber mats exhibit a more significant reduction in the ratio of the carbonyl peaks (*A*_1730_/*A*_1703_) as compared to that in the coated PU solid film. Additionally, the peak at 1685 cm^–1^ is distinctly pronounced in the spectra of the coated fiber mats as compared to the solid film. This suggests more pronounced interactions between the conductive coating and the substrates. Compared to the one-side-coated PU solid films, the electrospun 3D PU fiber mats have larger interfaces with the top PPy coating, resulting in more hydrogen bonding. This is evidenced by a consistent reduction in the ratio of the carbonyl peak areas (*A*_1730_/*A*_1703_) and a more pronounced peak at 1685 cm^–1^. This implies that the degree of interaction between the substrate and the coating via hydrogen bonding depends on the underlying substrate architecture and the oCVD PPy content.

### 2.3 XPS Analysis of oCVD PPy-Coated PU Films

The XPS analysis of samples with different oCVD PPy deposition times was performed to learn about the interactions between the PU fibers and the oCVD PPy coating. Figure S4 displays the XPS survey spectrum of the pristine PU fibers, which exhibit the signatures of the constituent elements of PU, namely carbon, nitrogen, and oxygen.

After oCVD PPy coating on the PU fibers, the survey spectra revealed the presence of Cl and Sb, in addition to C, N, and O (Figure S4). These findings are consistent with our previous study, which identified the presence of Sb and Cl in the PPy coating formed using the oCVD process.^34^Table S1 presents the results of a stoichiometric analysis that involved collecting detailed core level spectra of C 1s, N 1s, O 1s, Sb 3d, and Cl 2p for all samples and deducing the corresponding atomic percentages in the probed volume. As the deposition time increases, there is a corresponding increase in the atomic percentage of N and Cl, suggesting that a thicker coating of PPy is formed on the fibers.

**Figure 5:**
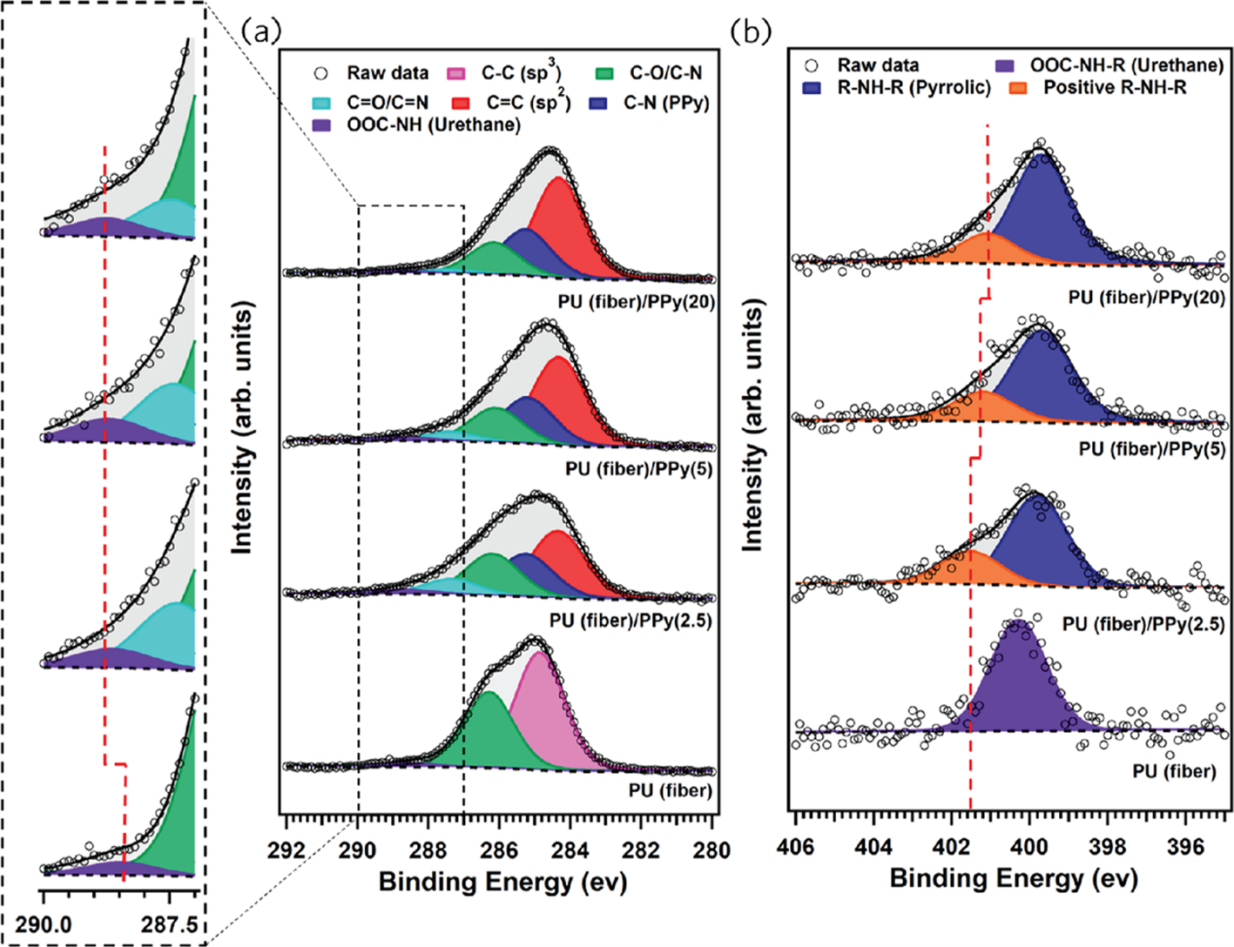
XPS spectra of (a) the C 1s core level region, with a magnified view shown in the dashed line box, and (b) N 1s core level region, both for pristine and PPy-coated PU fibers. The numbers 2.5, 5, and 20 in the file names stand for oCVD PPy deposition time in minutes.

Deconvolution of the C 1s core level spectrum of pristine PU fibers is shown in Figure 5a indicates the presence of three chemical species; the most significant component to the C 1s line at a binding energy (BE) of 284.8 eV (indicated in pink in Figure 5a) and accounting for ∼59.6% of the spectral intensity is attributed to sp^3^ C–C species.^50^ The components at BEs of 286.2 eV (indicated in green) and 288.4 eV (indicated in purple), respectively, represent C–O/C–N bonds (relative spectral intensity 38.6%) and OOC–NH of the urethane linkages^49^ (relative spectral intensity 1.8%). Applying the PPy coating on the PU fibers results in a noticeable alteration of the C 1s core level spectrum: the line becomes asymmetric and requires five components for an accurate fit. The peak marked in red at a BE of 284.2 eV is attributed to sp^2^ C=C species of oCVD PPy, while the other components situated at BEs of 285.1, 286.1, 287.3, and 288.8 eV are assigned to C–N (in PPy blue), C–O/C–N (green), C=O/C=N (cyan), and OOC–NH (purple) bonds, respectively.^34,51^ The component corresponding to urethane clearly shifts from a BE of 288.4 eV for pristine PU fibers to 288.8 eV after the PPy coating is applied, as indicated by the dashed line in the magnified view. Such a shift implies that the urethane linkages in the PU fibers are in a more positively charged chemical environment due to hydrogen bonding between the carbonyl groups of the urethane linkages and the −NH– species of the PPy coating. In this bonding, the carbonyl groups act as proton acceptors, while the −NH– species act as proton donors.^52^ The N 1s core level spectrum of pristine PU fibers displays a symmetric peak at a BE of 400.3 eV, corresponding to the N atoms present in the urethane bonds (Figure 5b).^53,54^ After applying PPy on the PU fibers, the N 1s core level spectrum becomes asymmetric, indicating the presence of two components. The main peak, marked in blue and located at a BE of 399.7 eV, is attributed to the −NH– species in the pyrrole ring, while the signature of the N atoms in the urethane linkages is no longer distinguishable due to overlap with the pyrrolic nitrogen species. The second component (marked in orange), with a BE in the range 401.1–401.5 eV, is associated with a positively charged nitrogen species resulting from the doping process.^34^ As shown in Figure 5b, the peak associated with positively charged nitrogen shifts to a lower BE as the oCVD PPy deposition time increases, from 401.5 eV in PU(fiber)/PPy(2.5) and to 401.1 eV in PU(fiber)/PPy(20). This shift mirrors the proton donation to the carbonyl groups of the urethane linkage in the PU fibers through hydrogen bonding, which results in partial charge compensation of the positively charged nitrogen species. This XPS analysis confirms the occurrence of hydrogen bonding between urethane bonds of PU fibers and positively charged nitrogen species in the oCVD PPy coating, in agreement with the results of the FTIR analysis.

### 2.4 Thermal Analysis of oCVD PPy-Coated PU Films

**Figure 6:**
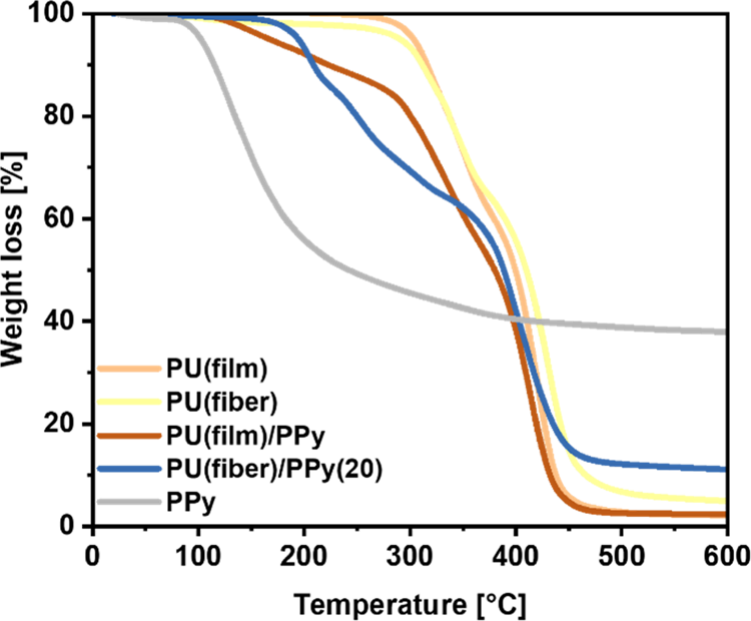
TGA analysis of the oCVD PPy-coated PU solid films (film) and electrospun fiber mats (fiber) as compared to their uncoated counterparts and pure PPy.

The thermal stabilities of the oCVD PPy-coated PU samples (film, fiber) were investigated by TGA, as illustrated in Figure 6. Pristine PU (film, fiber) and pure oCVD PPy were used for comparison. The thermograms of the pristine PU (film, fiber) exhibit thermal stabilities until approximately 290 °C, after which it undergoes a two-step degradation process. The first decomposition step records a peak loss at around 320 °C, corresponding to the hard urethane segments. The second peak loss is registered at 420 °C, which is attributed to the soft segments in the PU matrix.^55^ The thermogram of pure PPy shows that there is a mass loss at about 130 °C. This is ascribed to the de-doping of PPy due to the loss of volatile chloride dopant molecules upon heating and the short-chained PPy oligomers.^56^ The undoped PPy is thereafter thermally stable until temperatures exceed 350 °C. The onset of decomposition of oCVD PPy-coated PU solid films is recorded at approximately 167 °C, where it undergoes de-doping, followed by the two-step degradation of the PU chains. The thermograms of the oCVD PPy-coated electrospun PU fiber mats (Figures 6 and S6) show improved thermal stabilities up to 190 °C. After that, they exhibit similar decomposition steps of de-doping the PPy chains, followed by the two-step degradation of the PU matrix, identical to the oCVD PPy-coated PU solid films. The evidence suggests that the thermal stability of oCVD PPy at lower temperatures is improved upon coating on PU. This might be due to the molecular interactions between the underlying PU substrate and the oCVD PPy coatings via hydrogen bonding, as described earlier. The high extent of interactions in the oCVD PPy-coated electrospun PU fiber mats leads to much enhanced thermal stabilities at lower temperatures as compared to the one-side-coated PU solid films. The approximate weight percentage of oCVD PPy can be calculated from the residual weight of the samples and is summarized in Table 3.^57^ The absolute value of oCVD PPy content in the PU solid film is very low. However, optical microscopy images and the final electrical resistance of the samples provide evidence of significant and conformal deposition of a conductive coating layer on the surface.

**Table 3:** Summary of *T*_g_ Values of the Uncoated and oCVD PPy-Coated PU Samples with Their Corresponding PPy Content (Weight Percentage)

samples	oCVD PPy (weight %)	*T*_g_ (°C)
PU(film)	0	–52
PU(fiber)	0	–61
PU(film)/PPy	0.2	–50
PU(fiber)/PPy(2.5)	3.9	–58
PU(fiber)/PPy(5)	4.2	–57
PU(fiber)/PPy(10)	4.4	–56
PU(fiber)/PPy(20)	6.2	–54

Furthermore, DSC analysis was performed on the uncoated and the oCVD PPy-coated PU samples (film, fiber) to compare the glass-transition temperatures (*T*_g_) (Figure S5). All the samples undergo a glass transition within the range of −80 to −40 °C (Table 3). Both the oCVD PPy-coated solid films and the electrospun fiber mats show a higher *T*_g_ than their pristine counterparts. Such an increase in *T*_g_ became more pronounced for samples with higher PPy content. It is well known that the phase transition from the glassy to the rubbery state of PUs occurs due to increased mobility of the hard segments.^48^ Therefore, the shift of the *T*_g_ is attributed to the inclusion of the stiff oCVD PPy that is uniformly coated on the flexible PU substrates and their interactions via hydrogen bonding. Consequently, a higher amount of energy is required for the segmental molecular motion of the oCVD PPy-coated PU polymer chains in both the solid films and the electrospun fiber mats. This further corroborates our previous observations concerning the interactions between the oCVD PPy coating and the underlying PU substrates at a molecular level. Previous research on PPy-reinforced PU composites has reported similar observations regarding the shift of *T*_g_ to higher values. Yanilmaz et al. reported a shift of *T*_g_ of directly electrospun PU/PPy composite fibers to higher values than the pristine PU fiber mats. They explained it by the intermolecular interaction between the two components, which helped improve the phase mixing of the two components in the electrospinning solution.^46,48^

### 2.5 Mechanical Properties of oCVD PPy-Coated PU Films

**Figure 7:**
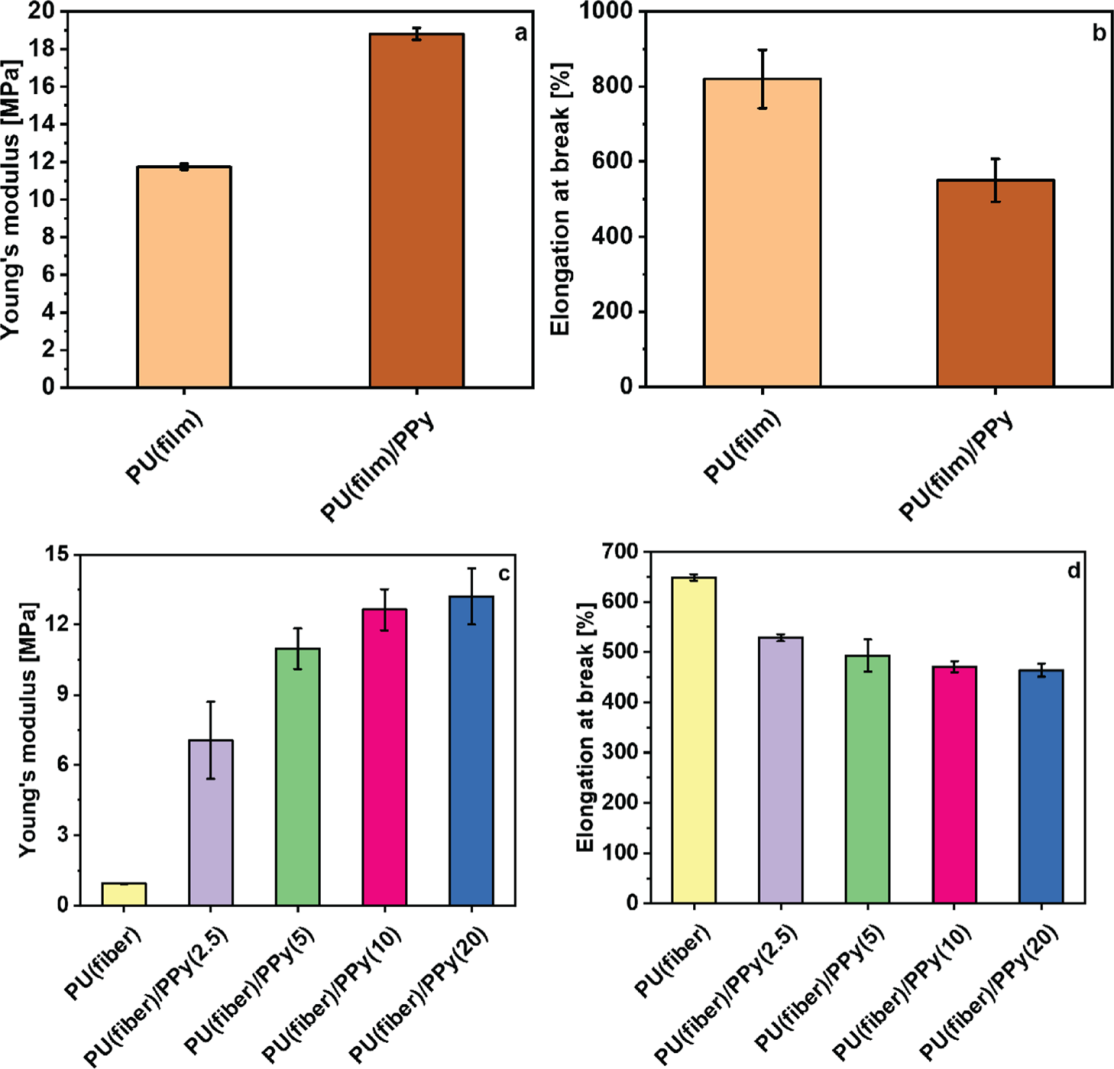
Comparison of Young’s modulus and elongation at break (%) of the uncoated and the oCVD PPy-coated PU solid films (film) (a,b) and electrospun PU fiber mats (fiber) (c,d).

The effect of the conductive oCVD PPy coating on the mechanical properties of the substrates was investigated by performing tensile tests on both the coated and uncoated PU samples (film, fiber), as shown in the stress–strain curves in Figure S7a,b. The corresponding Young’s moduli and elongation at break are compared in Figure 7a–d. The uncoated PU bulk films and the electrospun fiber mats are highly stretchable and elastic. The tensile curves of the uncoated samples shown in Figure S7a,b are sigmoidal in shape, characteristic of elastomeric materials.^58^ The tensile strength of the solid PU film is almost 1 order of magnitude higher than the value for the electrospun fiber mats, which can be attributed to the morphology of the two different samples. The electrospun fiber mats are highly porous; hence, the low material density affects the mechanical properties. The solid PU films exhibit a much higher Young’s modulus and tensile strength and a higher elongation at break than the electrospun fiber mats. After applying the oCVD PPy coating, the mechanical properties of the underlying substrates are not entirely compromised (Figure S7a,b), as they become stiffer but remain stretchable to an appreciable extent. This can be attributed to the ultrathin oCVD PPy coatings, which are thin enough to render the substrates stretchable. A similar trend is observed for the coated electrospun fiber mats. As the thickness of the oCVD PPy coating increased, the electrospun fiber mats became consistently stiffer, as shown by the increase in Young’s modulus. At the same time, the elongation strain at break decreased, which is attributed to the effect of the mechanically rigid oCVD PPy coating.^59^

**Figure 8:**
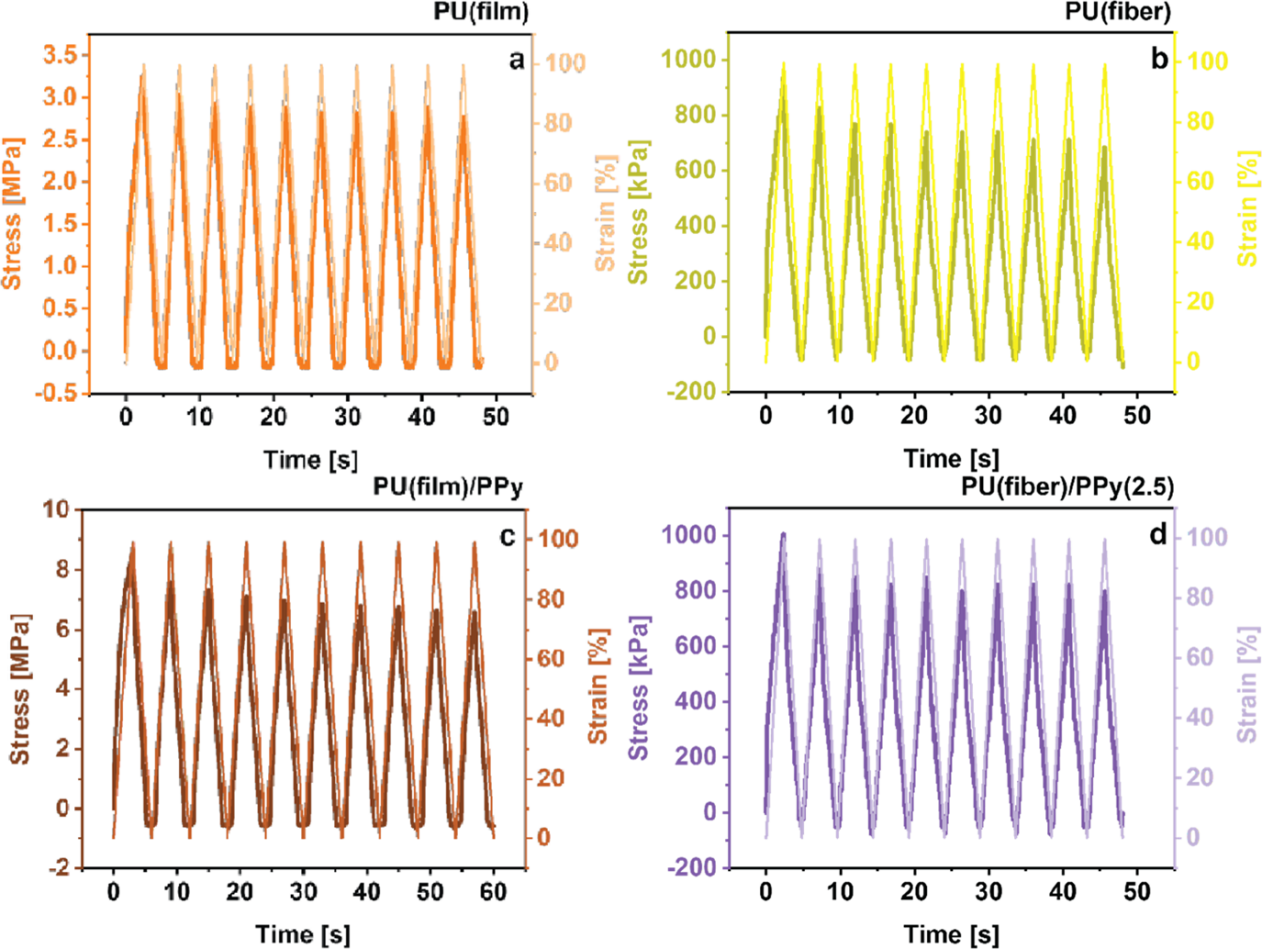
Cyclic stress response to 100% applied strain for 10 consecutive cycles for uncoated PU (a) solid films and (b) electrospun fiber mats, as compared to their oCVD PPy-coated counterparts (c,d).

The cyclic stress–strain curves of the pristine and the oCVD PPy-coated PU solid films and fiber mats are shown in Figures 8 and S8. As expected, both the pristine PU samples exhibited high degrees of stretchability and elasticity. As a result, they could rapidly recover their original shape without undergoing structural collapse or damage over 10 consecutive uniaxial stretching cycles of 100% applied strain. Upon loading, the stress in the PU film increased to ≈3 and ≈1 MPa for the electrospun PU fiber mats, which decreased sharply upon unloading. This resulted in a hysteresis loop in each stress–strain curve, indicative of the energy dissipation due to the viscoelastic nature of thermoplastic PU, as investigated in previous studies.^60,61^ On the other hand, the one-side-coated PU solid film accommodated much higher stress ≈8.5 MPa but exhibited irreversible deformation over the consecutive stretching cycles that can be attributed to the stiff oCVD PPy coating. The coated electrospun fiber mats showed a similar trend. As the oCVD PPy coating thickness increased, the fiber mats consistently accommodated higher stress within the material. However, compared to the PU solid films, the oCVD PPy-coated fiber mats exhibited much lower degrees of plastic deformation due to the ultra-thin coatings.

**Figure 9:**
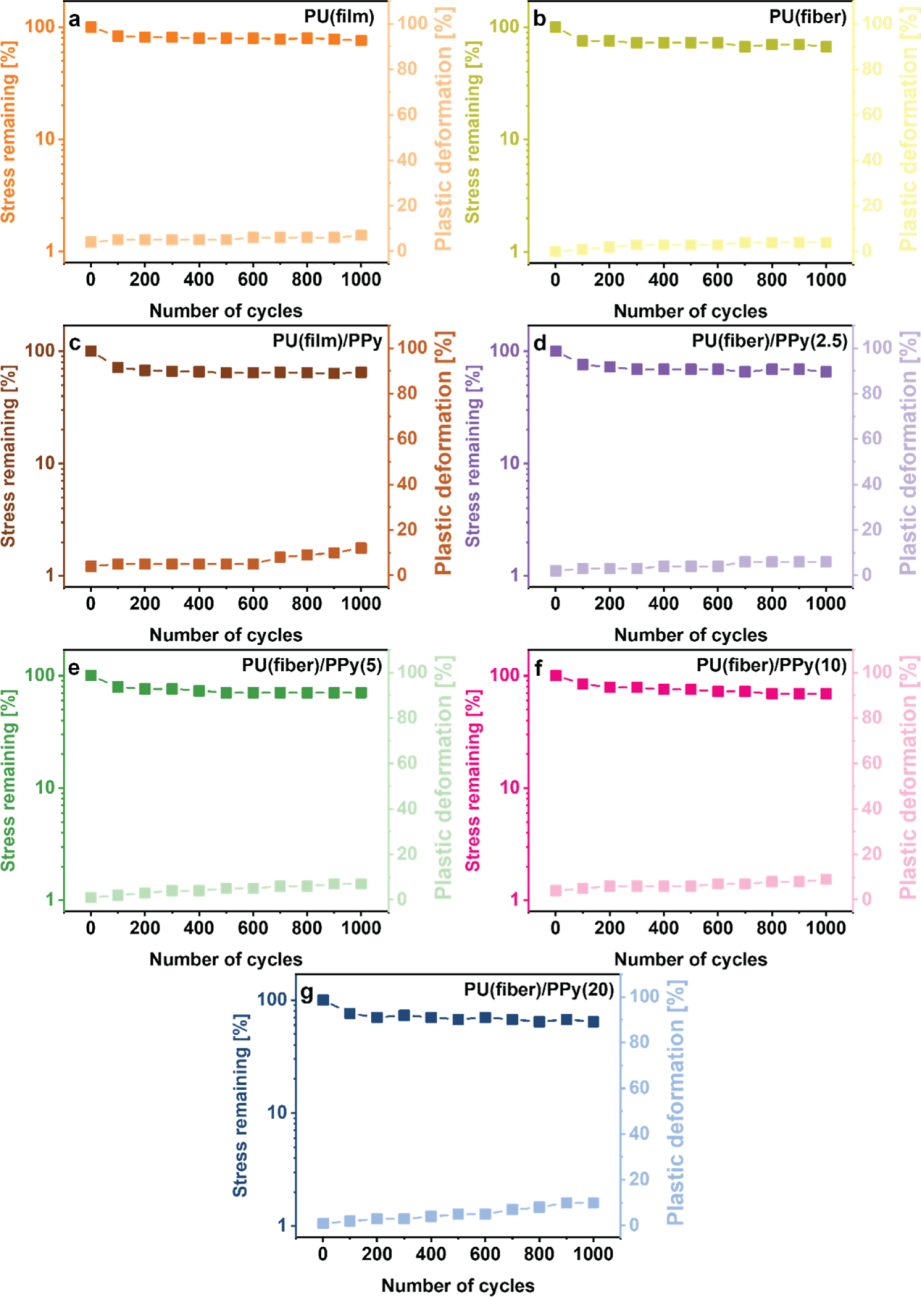
Change in maximum stress and plastic deformation over 1000 consecutive cycles of 100% applied strain showing fatigue resistance of oCVD PPy-coated (c) PU solid films (film) and PU fiber mats (fiber) for (d) 2.5; (e) 5; (f) 10; and (g) 20 min. Response of the pristine counterparts (a) PU (film) and (b) PU (fiber) has been used for comparison.

Furthermore, the fatigue behavior of the oCVD PPy-coated samples was investigated by stretching the samples over multiple cycles at different strains in succession. Figure 9 shows the excellent fatigue resistance of the electrically conductive oCVD PPy-coated samples to 100% applied strain over 1000 consecutive stretching cycles. The fatigue resistance of the samples to 25 and 50% applied strain over 1000 cycles in succession is illustrated in Figures S9 and S10, respectively. After 1000 stretching cycles at 25% applied strain, the oCVD PPy-coated PU solid film could accommodate ≈81% of the original stress while undergoing 6% plastic deformation. The electrospun fiber mats with the thinnest PPy coating showed even better fatigue resistance by adapting almost 95% of the original stress while registering only 3% plastic deformation at the end of 1000 cycles. In contrast, with continuous stretching cycles at 50% strain, the oCVD PPy-coated PU solid films accommodated almost 75% of the original stress with 7% plastic deformation. At the same time, the oCVD PPy electrospun fiber mat with the thinnest coating could still accommodate almost 93% of the maximum stress while undergoing 4% plastic deformation at the end of 1000 consecutive cycles with no resting time. It can be observed that both an increase in oCVD coating thickness and the applied strain resulted in a lowering of the maximum stress accommodated within the material and a slight increase in the plastic deformation. However, even with a substantial deformation of 100% strain for 1000 successive cycles, all the oCVD PPy-coated samples could accommodate 65% of the original stress. The lowering of the peak stress can be attributed to the mechanically rigid PPy coatings. The stiff PPy layers enhance the modulus of the elastic fibers due to which a high force is required to rearrange the fibers during the first cycles. However, the peak force is lowered after applying high percentages of dynamic strain due to microstructural defects in the mechanically rigid PPy coatings, especially for larger strains and higher coating thicknesses.^62^ It is essential to mention that even though the mechanical properties changed initially, they eventually stabilized during the subsequent stretching cycles. This suggests the samples could endure many cycles without experiencing additional fatigue damage.^63^ While oCVD PPy-coated solid films registered a plastic deformation of 12%, the electrospun fiber mats showed lower degrees of plastic deformation (6–10%) depending on the coating thickness, exhibiting good fatigue resistance.

### 2.6 Mechanoelectrical Properties of oCVD PPy-Coated PU Films

**Figure 10:**
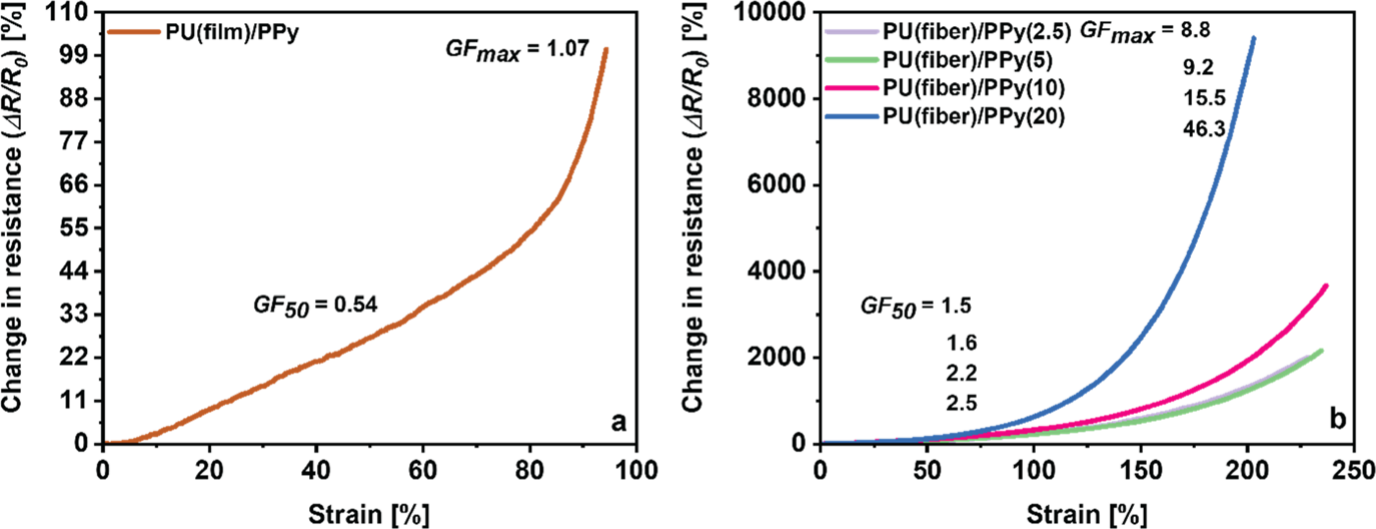
Change in resistance versus applied strain showing piezoresistivity of (a) the oCVD PPy-coated PU solid film (film) and (b) oCVD PPy-coated electrospun PU fiber mats (fiber) with increasing coating durations.

The mechanoelectrical properties of the oCVD PPy-coated PU (film, fiber) were investigated by monitoring the change in resistance of the material up to 10 MΩ while being exposed to uniaxial tensile strain. The normalized change in resistance of the electrically conductive samples is plotted against the applied mechanical strain (Figure 10a,b). Both the oCVD PPy-coated solid films and the electrospun fiber mats exhibit an appreciable change in resistance of the material over a broad range of structural deformation, indicating that the oCVD PPy PU (film, fiber) nanocomposites are piezoresistive. The piezoresistivity of the nanocomposites can be attributed to the microstructural changes in the oCVD PPy layer upon stretching. The deformation of the conductive oCVD PPy network elicits a change in the electrical resistance of the material as a function of the applied strain. As the films are stretched, the electron conduction pathway throughout the material is elongated, leading to a noticeable increase in electrical resistance.^64^ Both the oCVD PPy-coated PU (film, fiber) exhibited high degrees of stretchability and remained electrically conductive over a large strain regime (>100%). The oCVD PPy-coated PU (film) registers a maximum of 110% change in resistance in response to 94% applied strain. On the contrary, all the oCVD PPy-coated PU (fiber) exhibits a more significant change in resistance in response to the applied strain. The piezoresistive behavior of the oCVD PPy-coated fiber mats was investigated for different coating thicknesses. It can be observed that even the oCVD PPy PU (fiber) with the thickest coating preserved its electrical conductivity for large percentages of strain (>200%), which can be attributed to the strong interfacial adhesion between the two components as explored in the earlier sections. Compared to previous investigations, the oCVD PPy-coated nanocomposites provide a large working range and a highly sensitive piezoresistive response.^65−67^ As the oCVD PPy coating thickness increased, the piezoresistive response of the PU (fiber) was enhanced, as indicated by the slope of the curves in Figure 8a,b. The figure of merit for comparing the sensitivities of piezoresistive responses of electrically conductive materials is known as the gauge factor. The gauge factor (*GF*) is the normalized change in the electrical resistance of a material with respect to the applied strain.^68^
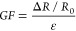
1where Δ*R* is the change in the resistance of the material, *R*_0_ is the initial resistance of the material, and ε is the strain applied to the material.

The *GF*s of both the PU (film, fiber) at 50% strain (*GF*_50_) as well as the maximum strain (*GF*_max_) enumerated from eq 1 are summarized in Table 4. As indicated by the slope of the curves, all the oCVD PPy-coated PU (fiber) recorded higher *GF* both at 50% strain as well as at maximum strain as compared to the coated PU (film). Interestingly, in the case of the oCVD PPy-coated PU (fiber), the samples with thicker conductive coatings exhibited higher *GF*. This can be attributed to the robust multiscale interfaces facilitated by the high surface area of the porous PU fiber mats and the conductive oCVD PPy coating. This allows for a more effective load transfer to the active oCVD PPy sensing layer, resulting in a repeatable piezoresistive response with higher sensitivity upon stretching. Furthermore, it is visible from Figure 10a,b that the piezoresistive response from the oCVD PPy-coated samples exhibited a linear change in the resistance of the material in the lower strain regime (<50%). In contrast, this behavior became nonlinear in the higher strain regimes.

**Table 4:** Summary of the Relative Change in Electrical Resistance with Respect to an Applied Strain Known as *GF* of oCVD PPy-Coated PU Solid Films (Film) and Electrospun Fiber Mats (Fiber) for 50% Strain (*GF*_50_) and Maximum Strain (*GF*_max_)

samples	electrical resistance (kΩ)	*GF*_50_	*GF*_max_
PU(film)/PPy	7	0.5	1.1
PU(fiber)/PPy(2.5)	473	1.5	8.8
PU(fiber)/PPy(5)	442	1.6	9.2
PU(fiber)/PPy(10)	267	2.2	15.5
PU(fiber)/PPy(20)	106	2.5	46.3

**Figure 11:**
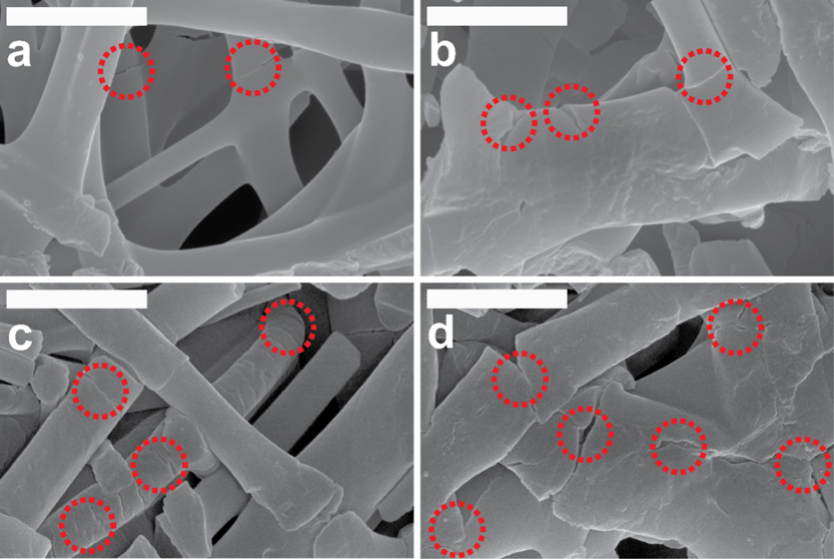
Morphology of the oCVD PPy-coated fiber mats for (a) 2.5, (b) 5, (c) 10, and (d) 20 min under 150% elongational strain showing distinct nano-cracks (marked in red) along the electrically conductive layer along the fibers. With increasing coating thickness, the fiber mats are visibly more prone to cracking under similar strained conditions. The scale bar for all SEM images is 1 μm.

To explore this further, a numerical curve fitting was performed on the piezoresistive curves shown in Figure S11a–e. The piezoresistive curves from all the oCVD PPy-coated samples are characterized by a linear response in the lower strain regime, exhibiting small changes in resistance. This linear behavior in the piezoresistive response curves can be attributed to the change in resistance of the material caused by dimensional changes. It can be observed from Figure S11a–e that this linear regime in the oCVD PPy-coated PU (film) is limited to 5% applied strain. In the case of the oCVD PPy-coated PU (fiber), the linear regime is extended to larger values of applied strain (≈50% elongation). The linear regime in the piezoresistive curve, caused by dimensional changes in the material, allows for repetitive cyclic strain sensing.^69,70^ For the oCVD PPy-coated PU (fiber), the linear regime is almost 10 times larger as compared to the PU (film). This indicates that the choice of substrate morphology allows for a tunable response at different strains. Figure S12a–e show the variation in the electrical resistance of oCVD PPy-coated PU (film and fiber) upon being subjected to 50% elongational strain through repeated cycles of stretching. The results demonstrate that, despite the increase in resistance during stretching for all samples, the resistance returned upon release, suggesting that the oCVD PPy-coated samples can detect piezoresistive changes repeatedly. Moreover, it is apparent that the change in electrical resistance during the stretching and releasing cycles is slightly different from that of the subsequent cycle. The drift in the change of resistance during this regime, mainly controlled by dimensional changes, can be attributed to the irreversible mechanical deformation during the stretching cycles. However, this effect is much less pronounced in the oCVD PPy-coated electrospun fiber mats than in the PU solid films. After that, an exponential regime of the piezoresistive curve ensues, characterized by very high degrees of change in resistance with respect to the strain applied. This exponential regime can be attributed to the thin oCVD PPy coatings cracking on the PU (film, fiber). Figure 11a–d display the morphology of the oCVD PPy-coated electrospun fiber mats under 150% elongational strain. The outer oCVD PPy coating on the fibers exhibits nano-cracks approximately 20 nm wide along the width of the fibers, despite being adhered. The electrically conductive oCVD PPy layer covers the entire surface of the electrospun fiber mats without stretching. Under significant elongational strain, the mechanically rigid PPy layer undergoes cracking, mostly concentrated around the nodes. As the material stretches further, the cracks in the electrically conductive oCVD PPy coatings widen, leading to an exponential change in the electrical resistance of the material due to an increase in the contact resistance.^62^ Previous research has shown that thin film piezoresistive sensors based on crack evolution result in a higher GF, as they exhibit a relatively more significant change in resistance with applied strains.^69^ In the case of oCVD PPy-coated PU (film), the exponential increase occurs at much lower strain percentages than the PU (fiber). Compared to the PU (film), the oCVD PPy-coated PU (fiber) exhibits a higher degree of interaction between the brittle oCVD PPy coating and the substrates; hence, it can be stretched to a greater extent. Interestingly, in the case of the PU (fiber), it can be observed that as the coating thickness increases, the exponential increase occurs at lower percentages of applied strain. This can be explained by the fact that thicker oCVD PPy coatings are more prone to microcrack evolution at low degrees of stretching, as is evident from Figure 11a–d. Moreover, it has also been reported that upon stretching, the porous PU (fiber) dissipates the stress by transferring it to the brittle PPy layer, owing to its strong interfacial adhesion.^70,71^ Hence, with increasing coating thickness, the stress distribution in the material is no longer uniform, leading to the cracking of the conductive coatings. Wang et al. also reported that thicker coating layers readily have microscopic defects in the structure, which causes early fracturing by applied stress.^72^

### 2.7 HDF Cell Culture Studies on oCVD PPy

The biocompatibility of PPy coatings was tested through the culture of HDF cells on top of the coated surfaces of 48 and 96-well plates. The performance of the cells was monitored on days 1, 4, and 7.

#### 2.7.1 Cell Viability

**Figure 12:**
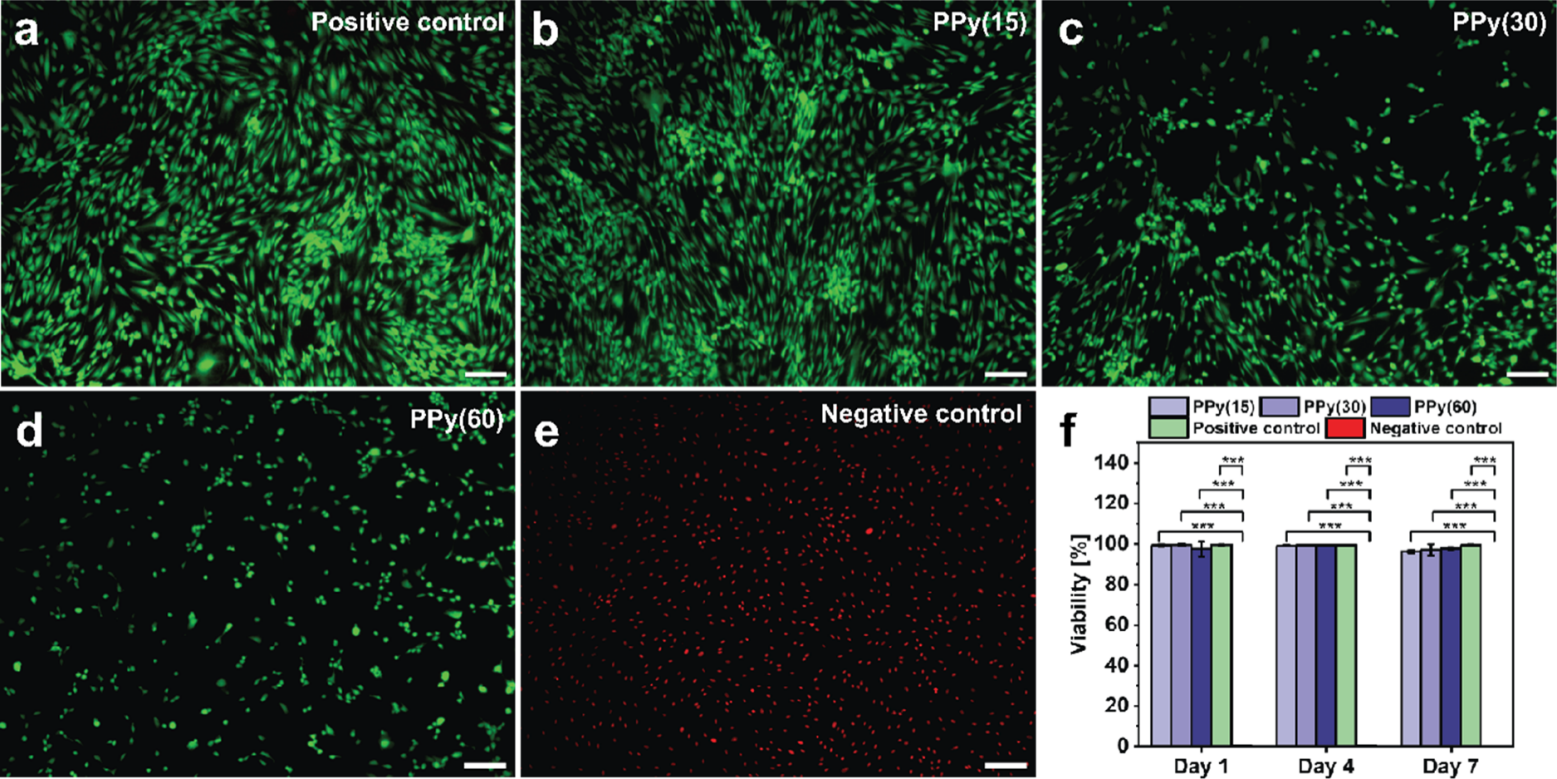
Fluorescence microscopy images of HDF cells cultured for 7 days and stained with FDA (green, living cells) and PI (red, dead cells) on (a) an uncoated surface serving as the positive control, a oCVD PPy-coated surface for (b) 15, (c) 30, and (d) 60 min, and (e) an uncoated surface treated with 70% ethanol prior to staining for the negative control. The scale bar for all images is 200 μm. (f) HDF cell viability on oCVD PPy-coated surfaces as compared to the positive and negative control over 7 days.

**Figure 13:**
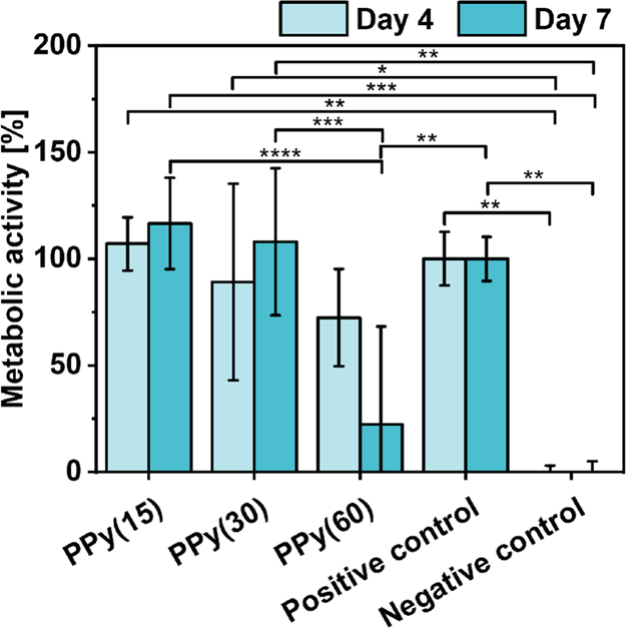
Metabolic activity of cells cultured on PPy-coated surfaces in comparison with negative (70% ethanol-treated) and positive (no coating) controls. The names PPy(15), PPy(30), and PPy(60) correspond to the oCVD PPy-coated surface for 15, 30, and 60 min, respectively.

The cell viability assessed via a live/dead assay oscillated around 100% for all coating thicknesses. It was maintained throughout 7 days of the experiment (Figure 12), indicating a lack of toxicity of the coatings. Although the viability for all the coating thicknesses was high, the number of cells observed for the thinnest PPy coating (PPy(15) ≈ 75 nm) was higher than that for the thicker PPy coatings (PPy(30) and PPy(60) ≈ 155 and 302 nm, respectively), as also confirmed by the MTT assay (see Figure 13). The overall cell number increases for all the coating types over the course of 7 days (see Figure S13). However, on day 4, there was a significant difference in cell number between the control and PPy(30) and PPy(60) coatings. Moreover, a trend can be observed, in which the cell number for PPy(15) is higher than for PPy(30), and PPy(60) has the lowest amount of cells, except for day 1. On day 7, the number of cells on PPy(15) surpassed the number registered for the control, although this difference does not bear statistical significance. The results are in line with the observations on the brightfield and fluorescence images, discussed in the later sections in detail. The lower number of cells present on the thicker PPy-coated wells could be attributed either to a slower growth rate on those coating thicknesses or could be caused by some of the cells dying and detaching from the surface during the washing steps.

#### 2.7.2 Cell Metabolic Activity

Cell metabolic activity on PPy-coated surfaces was calculated compared to negative (70% ethanol-treated) and positive (no coating) controls. The metabolic activity of HDFs on oCVD PPy-coated substrates and controls was quantified via the MTT assay (Figure 13). The cells cultured on PPy(15) and PPy(30) showed an increase in their metabolic activity between days 4 and 7, which indicates a possible increase in their proliferation. Interestingly, the cells grown on PPy(15) and PPy(30) showed significantly higher activity than the negative control on days 4 and 7, which shows that the culture conditions were favorable for the cells. Such a tendency was not observed for cells grown on PPy(60). Conversely, cells grown on this coating showed a decrease in their activity on day 7, and the values of metabolic activity for both time points were lower than those observed for the positive control. Together with the results from the live/dead assay test, where a smaller cell number is visible on those scaffolds (probably due to the washing of the dead cells), this observation indicates that the thickest (≈303 nm) PPy coatings can have a negative influence on the cells.

#### 2.7.3 Cell Morphology

**Figure 14:**
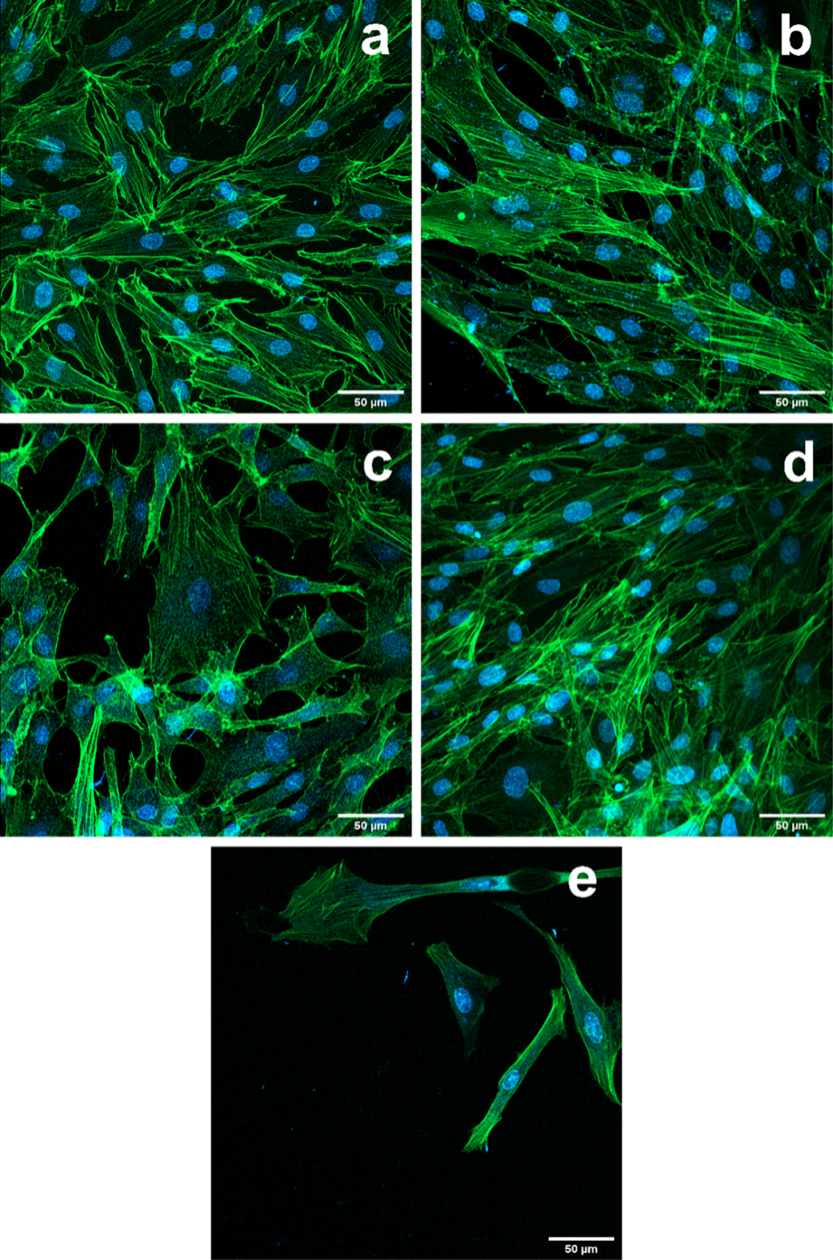
Fluorescence images of HDF cells stained with Hoechst nuclei staining (blue) and phalloidin actin staining (green). HDFs cultured for 7 days on the uncoated surface (a) and oCVD PPy-coated surface for (b) 15, (c) 30, and (d,e) 60 min (different spots on sample). The scale bar for all images is 50 μm.

The HDFs cultured on all PPy-coated surfaces of varying thicknesses adhere to the substrate and elongate (Figures S15 and S16). Cells on the thinner PPy-coated surface show better growth than on the thicker ones, as the number of cells visible on brightfield images resembles the number of cells of the control well and is higher than for the other two coating thicknesses. To further monitor cell morphology, F-actin deposition, and the nucleus shape of HDFs grown on coated surfaces, cells were stained with Hoechst and phalloidin. Fibroblasts are typically observed as spindle- or stellate-shaped cells with round nuclei. Such a spindle shape with an elongated cytoskeleton could be observed on all coating types and in the control wells on day 4 (Figure S14). More cells with a stellate shape can be seen on day 7 (Figure 14). The nuclei maintain their characteristic round shape and central position on days 4 and 7.

## 3 Conclusions

This work reports a vapor phase route toward synthesizing and characterizing stretchable and highly sensitive piezoresistive polymer nanocomposites with different polymer architectures. Ultrathin (5–102 nm) electrically conductive PPy coatings were realized via oCVD using antimony pentachloride as the oxidant on flexible PU bulk films and electrospun fiber mats. Peak deconvolution of the carbonyl peaks in the ATR spectra of the oCVD PPy-coated PU films revealed supramolecular interactions between the two components via hydrogen bonding, which was corroborated by XPS, TGA, and DSC of the nanocomposites as well. The electrospun PU (fiber) was highly porous and possessed a large surface-to-volume ratio, allowing for an enhanced degree of hydrogen bonding compared to the single-side-coated PU (film). Holding the porous PU (fiber) substrates perpendicular to the reactant flow as well as using a high patch flow of nitrogen during oCVD demonstrated improved mass transport as the reactant vapors were able to diffuse throughout the electrospun fiber mats and coat the individual fibers conformally with very thin conductive PPy coating without compromising the porous microstructure of the electrospun fiber mats and thus preserving the high surface to volume ratio. The mechanical properties of the resulting coated samples could be tuned with the thickness of the oCVD PPy coatings and showed excellent fatigue resistance to dynamic loading for upto 1000 consecutive cycles. The fabricated oCVD PPy-coated PU (film, fiber) was piezoresistive and showed repeatable changes in the electrical resistance of the material upon stretching. Thicker oCVD PPy coatings on the PU (fiber) enhanced the interfacial interactions and, consequently, the piezoresistive response due to effective load transfer to the active PPy layer when stretched, demonstrating GFs as high as ≈46 at 202% strain. In situ monitoring of the change in electrical resistance of the material with applied strain revealed two underlying regimes in the resulting piezoresistive response curves. The variation in resistance of the piezoresistive conductive polymer nanocomposites in the low strain regime is due to dimensional changes registered as low GFs. In contrast, the evolution of nano-cracks on the conductive oCVD PPy coating led to more significant variations in the high-strain regime, resulting in elevated GFs. The viability of HDF cells remained high across all coating types over the 7 day period, but thicker PPy coatings showed lower cell numbers, potentially due to toxicity, reduced proliferation, or cell removal. Cell morphology on all coated wells resembled dermal fibroblasts, and metabolic activity was similar to or higher than that of the positive control for thinner oCVD PPy coatings. Thinner coatings are thus preferred for future cell experiments, as they exhibit favorable metabolic activity and maintain cell morphology. Based on these results, the proposed fully polymer-based stretchable and biocompatible, electrically conductive nanocomposites can be used to design piezoresistive strain sensing elements for wearable and skin-conformable electronics for healthcare monitoring, involving both small and large strain-related dynamic movements.

## 4 Experimental Section/Methods

### 4.1 Materials

Thermoplastic PU (PU, Elastollan 1170 A10) was supplied by BASF, Germany. Dimethylformamide (DMF, HCON(CH_3_)_2_), tetrahydrofuran (THF, C_4_H_8_O), and antimony pentachloride (SbCl_5_, >98%) were purchased from Sigma-Aldrich. Pyrrole (Py, C_4_H_5_N, >99%) was obtained from TCI Europe N.V. All chemicals were used as received without further purification. IR transparent silicon wafers (single-side polished, prime CZ) were purchased from Sil’tronix, France.

### 4.2 Fabricating Flexible PU Substrates

Flexible PU substrates were developed as solid films by solvent casting and fiber mats by electrospinning (Figure 1). To produce the solid films, PU pellets were dissolved in THF (10% w/w) by stirring at room temperature for approximately 2 h. Thereafter, the precursor solution was cast onto a PTFE slab using a film applicator and left to dry overnight in the fume hood. The films were covered with aluminum foil with holes to control the drying process and prevent contamination. The final solid PU films, denoted as PU (film), had a dry thickness of approximately 100 μm.

For electrospinning fiber mats, PU pellets (10% w/w) were dissolved in a mixture of DMF and THF (1:3) and stirred overnight at 60 °C to obtain a colorless, homogeneous precursor solution. The PU fiber mats were electrospun using an Inovenso NanoSpinner NE300 electrospinning machine at ambient conditions. The precursor solution was transferred to a plastic syringe fitted with an 18 G needle and attached to a syringe pump. The volume flow rate of the syringe pump was set to 1 mL h^–1^. An aluminum foil-wrapped drum rotating @ 100 rpm was used as the collector. The tip-collector distance was maintained at 15 cm, while a voltage of 15 kV was applied. Electrospinning was performed until free-standing PU fiber mats (thickness ≈ 100 μm), hereafter denoted as PU (fiber), could be detached from the aluminum foil for further use.

### 4.3 oCVD of PPy on Various Substrates

oCVD of PPy was performed using vaporized Py monomer and antimony pentachloride as the oxidant in a custom-built CVD reactor, as shown in Figure 1. The reactant gas lines were always maintained at 110 °C to prevent condensation of the reactants, and the main reaction chamber was heated to 40 °C. Both reactants were heated in glass jars to ensure enough vapor pressure for maintaining constant flows during the reaction. The flows of the monomer and the oxidant were metered to a continuous flow of 2.5 and 0.5 sccm, respectively, using precision valves. The oxidant-to-monomer flow ratio was held at a constant value of 0.2 to prevent overoxidation and fed to the main reaction chamber via two separate inlet ports perpendicular to each other. A constant patch flow of nitrogen was used through the oxidant delivery gas line during the reaction as a diluent as well as to facilitate the flow of the oxidant. The reactions were performed at a constant pressure of 300 mTorr. PU (film) and PU (fiber) were used as substrates. Pure PPy coatings were obtained by performing similar reactions using IR transparent silicon wafers and glass slides as substrates and utilized for further characterization. Additionally, cell plates were coated with pure PPy by masking the appropriate sections for use as control and for cell culture studies. In all cases, the stage temperature was maintained at 40 °C. The reactions were performed for 30 min on PU (film) with 2 sccm of nitrogen patch flow and for 2.5/5/10/20 min with 30 sccm of nitrogen patch flow on PU (fiber) to achieve PPy coatings of different thicknesses. The cell plates were coated for 15/30/60 min with the same nitrogen patch flow. It should be noted that while the PU (film) was adhered to the base of the reactor and was coated only on one side, the PU (fiber) was attached to a custom-built stage and held perpendicular to the reactant gas flows, aligned with the monomer flow to reduce direct exposure with the oxidant and allow coating on both sides, simultaneously. All the samples were stored in an inert argon-filled chamber after oCVD.

### 4.4 Polymer Characterization

The morphology of the uncoated and the PPy-coated PU (fiber) was observed by SEM (Nova NanoSEM 650) with a working distance of 5 mm and an acceleration voltage of 10 kV. To avoid charging effects, the pristine PU (fiber) samples were coated with 10 nm of gold before making observations. The mean fiber diameter of the uncoated and oCVD PPy-coated PU fiber mats was calculated by performing a fiber diameter analysis (averaging over 100 fibers) using ImageJ software. The significance of the increase in mean fiber diameter in the oCVD PPy-coated PU fiber mats was evaluated by performing a *p*-test with a 95% confidence interval using GraphPad Prism software. The morphology of the oCVD PPy-coated PU(fiber) samples, pre-stretched to approximately 150% tensile strain, was observed by SEM with similar conditions.

The chemical composition of all the uncoated and the oCVD PPy-coated PU (film, fiber) was investigated by ATR (Shimadzu IRTracer) in absorbance mode. The spectra were collected over a wavenumber range of 800–3600 cm^–1^ with a resolution of 4 cm^–1^ and averaged over 128 scans. Pure PPy was investigated by performing FTIR on PPy-coated IR transparent silicon wafer in absorbance mode, keeping all other parameters constant, and using a bare silicon wafer as the background.

XPS was performed using a Surface Science SSX-100 ESCA instrument with a monochromatic Al Kα X-ray source (*h*ν = 1486.6 eV). The pressure in the analysis chamber was maintained below 5 × 10^–9^ mbar, the electron take-off angle was set to 37° with respect to the normal surface, and a spot with a diameter of 1000 μm was used to collect spectra. A gold grid was placed on the top of the samples, and a flood gun was employed to direct electrons with very low kinetic energy onto the sample during data acquisition. This was done to avoid charging effects that may occur during XPS measurements. For both the survey spectra and the detailed spectra of the C 1s, N 1s, Cl 2p, and Sb 3d core levels, the energy resolution was configured to 1.3 eV. In the case of pristine PU fibers, the binding energies were calibrated with reference to the sp^3^ C 1s photoemission peak located at a binding energy (BE) of 284.8 eV. For PPy-coated samples, the binding energies were referenced to the sp^2^ C 1s photoemission peak located at a BE of 284.2 eV. The reported binding energies are accurate ±0.1 eV. To analyze the XPS data, a least-squares curve-fitting program, Winspec (LISE laboratory, University of Namur, Belgium), was utilized. The analysis involved performing a Shirley baseline subtraction and fitting with a minimum number of peaks. The peak profiles were taken as a convolution of Gaussian and Lorentzian functions. To ensure the homogeneity of the samples, measurements were conducted at two different spots of each sample.

The thermal stability of all the uncoated and the oCVD PPy-coated PU (film, fiber) and pure PPy was investigated by TGA performed on a PerkinElmer TGA 7 from room temperature until 600 °C with a heating rate of 10 °C min^–1^ under nitrogen. DSC measurements were made using TA DSC Q1000 equipment to observe the *T*_g_ of the samples. The data were collected over a temperature range of −80 to 80 °C with a heating rate of 10 °C min^–1^. The thermal history of the samples was erased by exposing the samples to a heating and cooling cycle before data collection.

The tensile tests for the uncoated and the oCVD PPy-coated PU (film) were performed on an Instron 5565 tensile testing equipment with a constant cross-head speed of 50 mm min^–1^. The uncoated and oCVD PPy-coated PU (fiber) mechanical properties were evaluated by performing uniaxial tensile tests on the rheometer HR20 in tension mode with a constant cross-head speed of 5 μm s^–1^. The Young’s modulus and the elongation at break for all the samples were collected from the data. The cyclic tensile tests for the uncoated and the oCVD PPy-coated PU (film, fiber) were performed on a CellScale UniVert mechanical tester with a 10 N load cell. The fatigue behavior of the samples was evaluated by applying 1000 consecutive cycles of 25, 50, and 100% strain at a constant rate of 20 mm min^–1^, and the residual stress and plastic deformation over the different cycles were calculated from the data. The mechanoelectrical properties of the oCVD PPy-coated PU (film, fiber) in response to quasistatic tensile strain were investigated by attaching electrodes to the tension clamps in the rheometer HR20, which measured the applied strain and resulting stress over time with a constant cross-head speed of 50 μm s^–1^ in ambient conditions. Similarly, the cyclic piezoresistive response of the PU (fiber) was obtained in response to 10 consecutive cycles of 50% applied strain. The electrodes were wired on the other end to a modular digital multimeter (Keysight BenchVue U2741A), measuring the electrical resistance of the material over time with a sampling interval of 1 s. The electrical resistance of the samples in the unstrained state was evaluated by averaging resistance values over the first 3 min of the test.

### 4.5 Cell Culture of Human Dermal Fibroblasts on oCVD PPy

To perform the biocompatibility tests, oCVD PPy was deposited directly on cell culture plates (described in Section 4.3) for the following tests.

Primary HDFs derived from adult skin (P10856, Innoprot, Spain) were cultured according to the protocol from the provider. Briefly, the culture was set up in 2 μg cm^–2^ poly-l-lysine-coated T75 flasks (overnight) at a recommended seeding density of 5000 cells cm^–2^. DMEM (31966021, Gibco) supplemented with 10% fetal bovine serum (F9665, Sigma-Aldrich), 1% penicillin–streptomycin (10,000 U mL^–1^, 15140148, Gibco), and 1 ng mL^–1^ fibroblast growth factors (PHG0367, Gibco) was used as cell culture medium. HDF cells were cultured at 37 °C in a humidified atmosphere of 5% CO_2_. The medium was refreshed every 2–3 days until the cells reached over 90% confluency.

The PPy-coated well plates were sterilized before cell seeding by incubation in 70% for 2 h, followed by triple DPBS (D8537, Sigma-Aldrich) wash. After sterilization, each well was filled with a complete cell culture medium and kept at 37 °C until cell seeding. HDF cells at passage 5 were seeded in PPy-coated adherent 96- and 48-well plates (83.3924, Sarstedt, and 677180, Greiner Bio-One, respectively) at a recommended density of 5000 cells cm^–2^. The seeded cells were cultured for 7 days, with the cell culture medium changed every 2–3 days. Cells grown in 48-well plates on days 1, 4, and 7 were used for cell viability assays. Cells grown in 96-well plates on days 4 and 7 were used for metabolic activity assays, light microscopy observations, and immunofluorescence staining. Cells grown in non-coated wells in each well plate served as controls.

#### 4.5.1 Cell Viability

A live/dead assay was performed to determine cell viability on PPy-coating. Shortly, solutions of 20 μg mL^–1^ of fluorescein diacetate (FDA) (F1303, Thermo Fisher Scientific) in DPBS and 20 μg mL^–1^ propidium iodide (PI) (P1304MP, Thermo Fisher Scientific) in DPBS were prepared. Before performing the assay, half of the control samples were treated with 70% ethanol for 5 min, followed by a triple wash with DPBS. Next, the cell culture medium was removed from the remaining wells and exchanged with 100 μL of FDA and 30 μL of PI working solutions, followed by 10 min of incubation at room temperature. After a double DPBS wash of each well, the cells were imaged with an inverted fluorescence microscope (Leica DM IL Led). Ethanol-treated cells served as negative (dead) control, whereas cells grown in non-coated wells without ethanol treatment served as positive (live) control.

Six images per sample were used for analysis, except for negative controls on days 1 and 4, in which four images per sample were used. Each image was analyzed using Fiji software. First, brightness and contrast were adjusted and then the threshold. Subsequently, the functions “watershed” and “analyze particles” were used to determine the number of cells. Finally, cell viability was calculated via eq 2
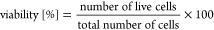
2

The number of cells calculated for cell viability studies was used to prepare the cell count graph.

#### 4.5.2 Cell Metabolic Activity

The cell metabolic activity assay was assessed via the MTT assay (475989, Sigma-Aldrich). Briefly, on days 4 and 7, cell medium was removed from the wells, and 100 μL of 0.5 mg mL^–1^ MTT solution was added into each well, followed by 1 h of incubation at 37 °C. After that, the solution was removed from the wells, and the plates were stored for at least 1 h in the freezer for further use. Subsequently, 60 μL of DMSO (41639, Sigma-Aldrich) was added into each well, and the plates were incubated for 1 h at 37 °C while shaking. Finally, the absorbance was measured at 570 and 630 nm with a hybrid multi-mode microplate reader (Synergy H1, BioTek Instruments) for background subtraction. Cells cultured on a non-coated substrate were positive controls, and cells treated with 70% ethanol were negative controls. Therefore, the metabolic activity was calculated from the following eq 3
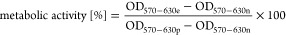
3where, OD_570–630e_ is the mean value of the optical density of the test samples, OD_570–630n_ is the mean value of the optical density of negative control, and OD_570–630p_ is the mean value of the optical density of positive control.

#### 4.5.3 Immunofluorescence

Immunofluorescence of nuclei and the cytoskeleton of the HDF cells were carried out on days 4 and 7. First, the cells were washed with DPBS and then fixed in 4% PFA for 15 min, following triple DPBS wash. Then, the cells were incubated for 1 h with a solution of 0.001% Tween 20 (P7949, Sigma-Aldrich) and 1:100 Alexa Fluor 488 phalloidin (A12379, Thermo Fisher Scientific) in DPBS. Wells were subsequently rinsed three times with DPBS and incubated for 5 min with 1:10,000 Hoechst (62249, Thermo Fisher Scientific) in DPBS. Imaging was performed with a confocal microscope (Zeiss Cell Discoverer 7).

#### 4.5.4 Statistics

All the calculated results are reported as the mean value ± standard deviation. Statistical significances were analyzed via one-way ANOVA or two-way ANOVA and a post hoc Turkey test using GraphPad Prism software. Differences with *p* < 0.05 were considered significant.

## Supporting Information Available

Fiber diameter analysis and test of significance; deposition rate of oCVD PPy; raw FTIR absorbance spectrum of oCVD PPy; red shift of hydrogen bonded −NH groups in PU (fiber) after oCVD PPy coating; peak deconvolutions of carbonyl functional peaks in the ATR spectra of oCVD PPy-coated PU (fiber) for different durations; XPS survey spectra for pristine and oCVD PPy-coated PU (fiber) samples; surface chemical composition of pristine and oCVD PPy-coated PU (fiber) samples as deduced from XPS; DSC curves of oCVD PPy-coated PU (film, fiber); TGA curves of oCVD PPy-coated PU (fiber) for different durations; stress–strain curves of oCVD PPy-coated PU (film, fiber) compared to their pristine counterparts; cyclic stress response to 100% applied strain for 10 consecutive cycles of oCVD PPy-coated PU (fiber) for different durations; fatigue resistance of uncoated and oCVD PPy-coated PU (film, fiber) over 1000 consecutive cycles of 50% applied strain; fatigue resistance of uncoated and oCVD PPy-coated PU (film, fiber) over 1000 consecutive cycles of 25% applied strain; numerical curve fitting of oCVD PPy-coated PU (film, fiber); cyclic piezoresistive response of oCVD PPy-coated PU(film, fiber) and corresponding GFs; cell count per well of HDF cells on oCVD PPy of different thicknesses calculated from fluorescence microscopy images over 7 days; fluorescence images of HDF cell cultures on oCVD PPy-coated surfaces for 4 days; bright-field images of HDF cell morphology cultured on oCVD PPy-coated surfaces of various thicknesses for 4 days; and bright-field images of HDF cell morphology cultured on oCVD PPy-coated surfaces of various thicknesses for 7 days (PDF)

## Abbreviations

ICPs: inherently conductive polymers
PPy: polypyrrole
PANI: polyaniline
PEDOT: poly(3,4-ethylene dioxythiophene)
oCVD: oxidative chemical vapor deposition
PU: polyurethane
SEM: scanning electron microscopy
FTIR: Fourier-transform infrared
ATR: attenuated total reflection
XPS: X-ray photoelectron spectroscopy
TGA: thermogravimetric analysis
DSC: differential scanning calorimetry
CNF: carbon nanofiber
GF: gauge factor
