# Supplementary material for: Electrically Conductive and Highly Stretchable Piezoresistive Polymer Nanocomposites via Oxidative Chemical Vapor Deposition
Source: ACS Appl Mater Interfaces. 2023 Jun 22;15(26):31899–916. doi: 10.1021/acsami.3c06015 (PMC10326852; doi:10.1021/acsami.3c06015)
Supplement: Supplementary file 1 — am3c06015_si_001.pdf [file am3c06015_si_001.pdf]

**Supporting Information**

# Electrically Conductive and Highly Stretchable Piezoresistive Polymer Nanocomposites via Oxidative Chemical Vapor Deposition

*Adrivit Mukherjee[†,□,□], Afshin Dianatdar[†], Magdalena Z. Gładysz[□], Hamoon Hemmatpour[†], Mart Hendriksen[†], Petra Rudolf[‡], Małgorzata K. Włodarczyk-Biegun[□], Marleen Kamperman[□], Ajay Giri Prakash Kottapalli[□]*, Ranjita K. Bose[†]**

[†]Chemical Product Engineering, Engineering and Technology Institute Groningen (ENTEG), University of Groningen, Nijenborgh 4, 9747 AG, The Netherlands

[□]Polymer Science, Zernike Institute for Advanced Materials (ZIAM), University of Groningen, Nijenborgh 4, 9747 AG, The Netherlands

[‡]Surfaces and Thin Films, Zernike Institute for Advanced Materials (ZIAM), University of Groningen, Nijenborgh 4, 9747 AG, The Netherlands

Advanced Production Engineering, Engineering and Technology Institute Groningen (ENTEG), University of Groningen, Nijenborgh 4, 9747 AG, The Netherlands

**Corresponding authors:**

†*Ranjita K. Bose - E-mail: r.k.bose@rug.nl

*Ajay Giri Prakash Kottapalli - Email: a.g.p.kottapalli@rug.nl

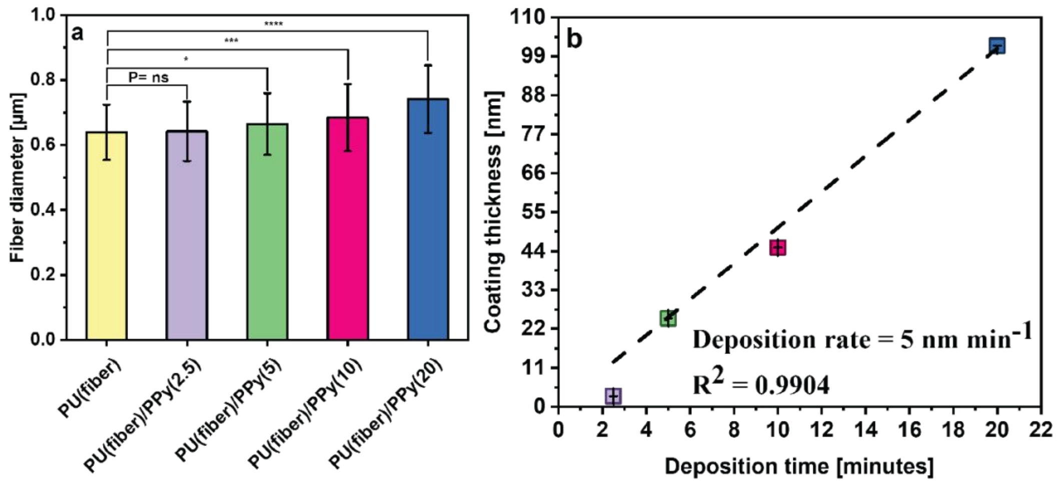


**Figure S1a.** Fiber diameter analysis of oCVD polypyrrole (PPy) coated PU electrospun fiber-mats (fiber) showed an increase in average fiber diameter with longer durations of deposition. A t-test has validated the statistical significance of the difference between the results; and **b.** the coating

thickness of oCVD polypyrrole (PPy) on the PU fiber-mats (fiber) was plotted as a function of deposition time, showing an average deposition rate of 5 nm $min^{-1}$.

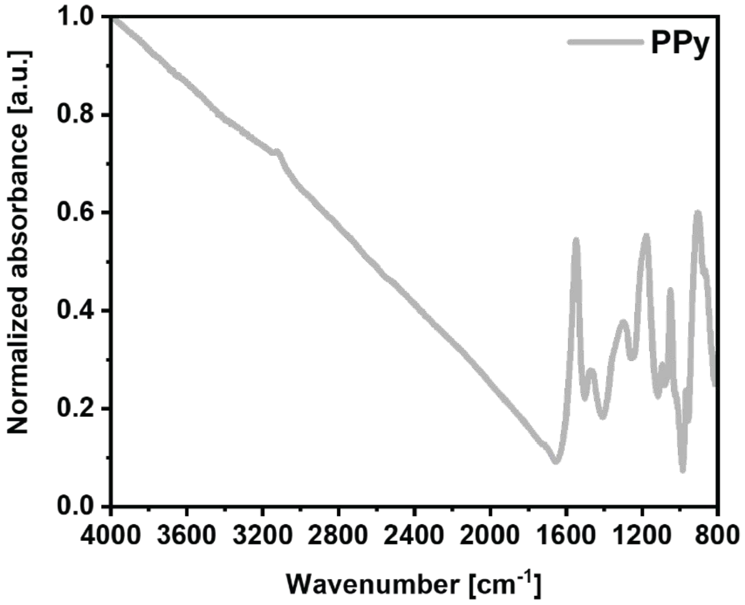


**Figure S2.** Baseline uncorrected normalized FTIR absorbance spectrum of as-deposited oCVD polypyrrole (PPy) on a silicon wafer showing characteristics of doped polypyrrole.

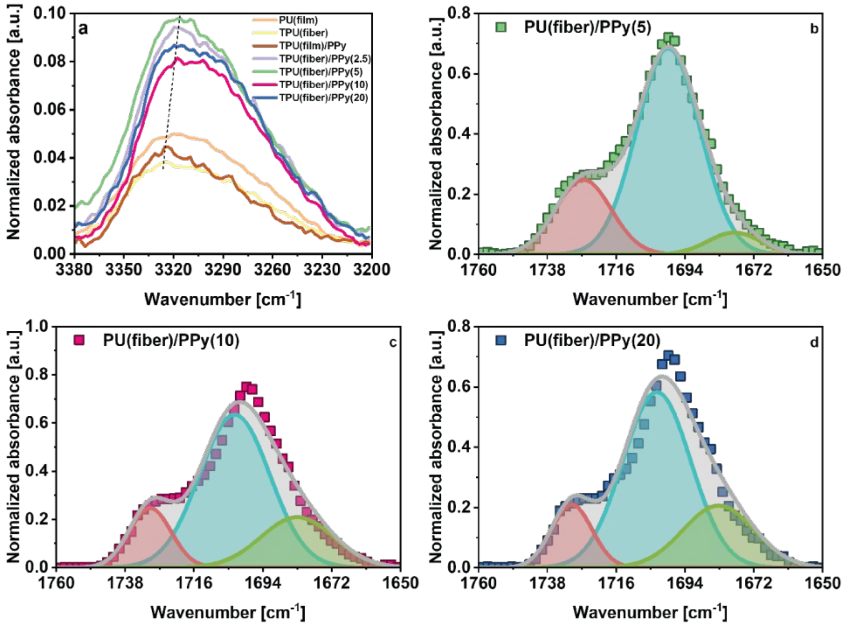


**Figure S3a.** The red shift of the hydrogen-bonded -NH stretching of the PU (film, fiber) after oCVD polypyrrole (PPy) coating; Peak deconvolutions of the carbonyl functional peaks in the ATR spectra of oCVD polypyrrole (PPy) coated electrospun PU fiber-mats for **b.** 5; **c.** 10 ; and **d.** 20 minutes, respectively.

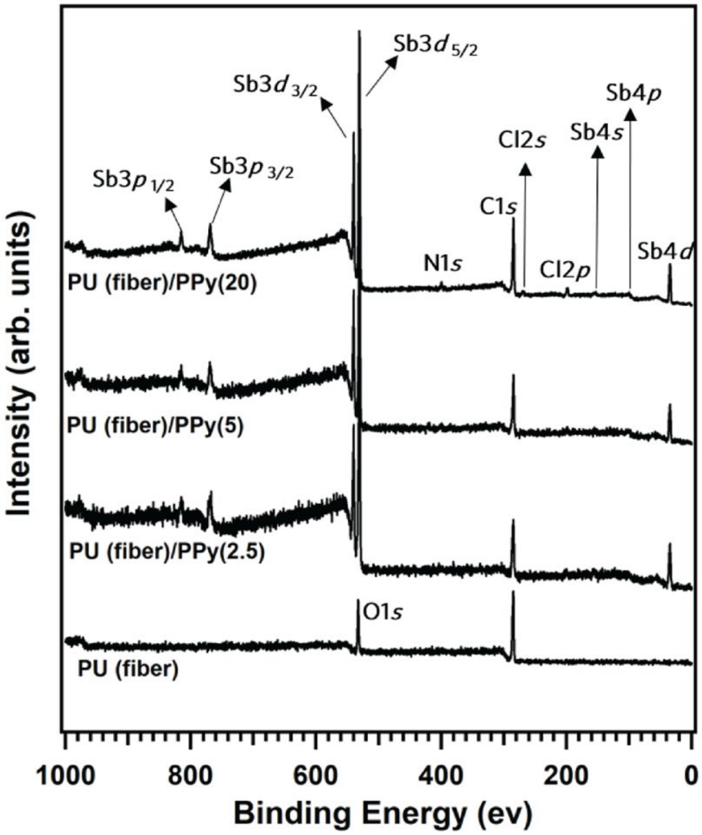


**Figure S4.** XPS survey spectra for pristine and oCVD polypyrrole (PPy) coated PU fiber-mats (fiber).

**Table S1.** Surface chemical composition of pristine and oCVD polypyrrole (PPy) coated PU fiber-mats (fiber), as deduced from XPS analysis.

| Sample | Atomic percentage (at. %) | | | | |
|---|---|---|---|---|---|
| | C | N | O | Sb | Cl |
| PU(film)/PPy | 76.5 | 1.8 | 21.7 | - | - |

| | | | | | |
|---|---|---|---|---|---|
| PU(fiber)/PPy(2.5) | 59.1 | 1.5 | 26.4 | 11.6 | 1.4 |
| PU(fiber)/PPy(5) | 64.3 | 2.7 | 21.4 | 10.0 | 1.6 |
| PU(fiber)/PPy(20) | 64.0 | 2.8 | 19.3 | 11.0 | 2.9 |

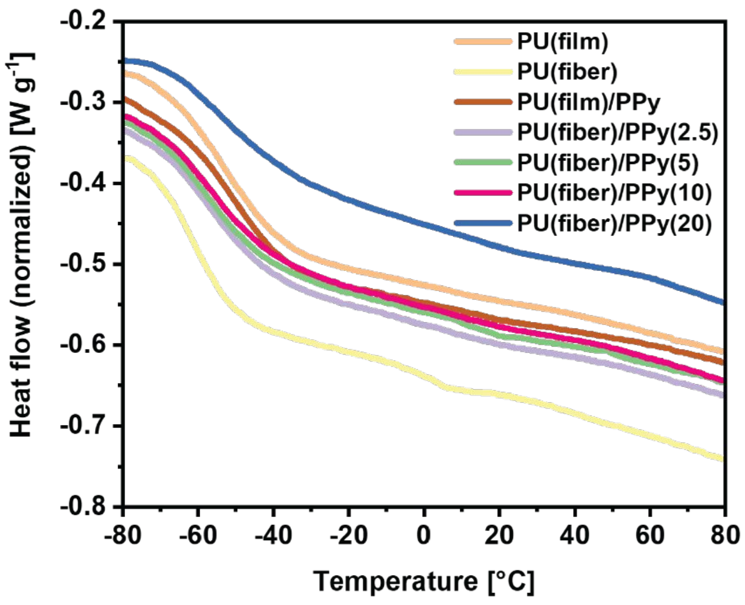


**Figure S5.** DSC curves of the one side oCVD polypyrrole (PPy) coated PU solid films (film) and electrospun PU fiber-mats (fiber) coated uniformly with oCVD PPy. The DSC curves of uncoated PU solid film (film) and electrospun fiber-mat (fiber) are used for comparison of the glass transition temperatures ($T_g$).

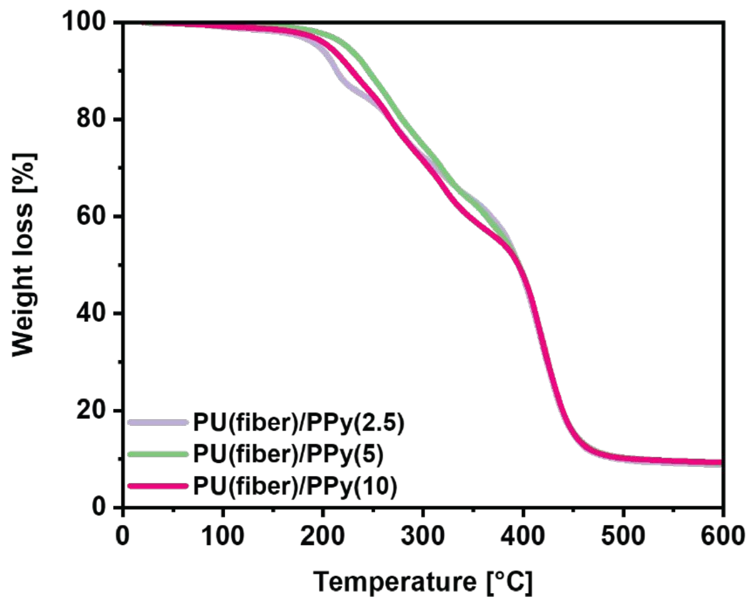


**Figure S6.** TGA of oCVD polypyrrole (PPy) coated electrospun fiber-mats (fiber) for 2.5/5/10 min.

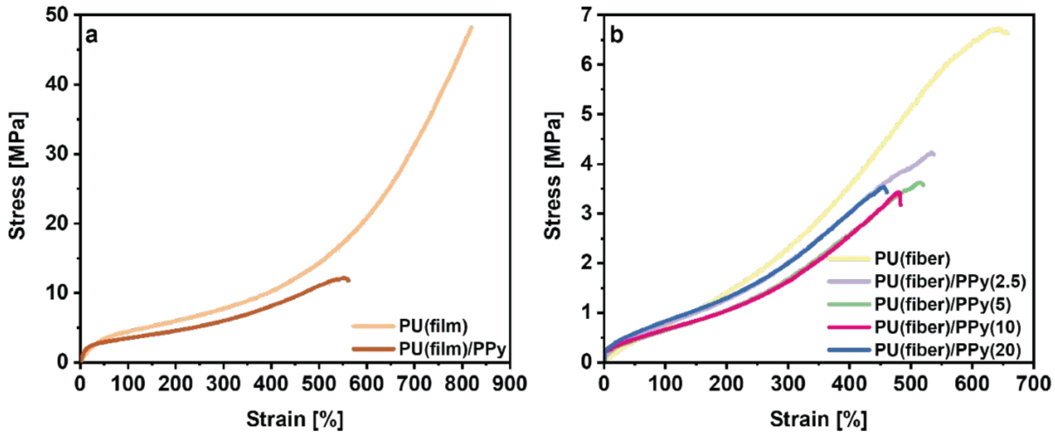


**Figure S7.** Representative stress-strain curves for uniaxial tensile tests of oCVD polypyrrole (PPy) coated: **a.** PU solid films (film); and **b.** electrospun PU fiber-mats (fiber) for different coating durations compared to their pristine counterparts.

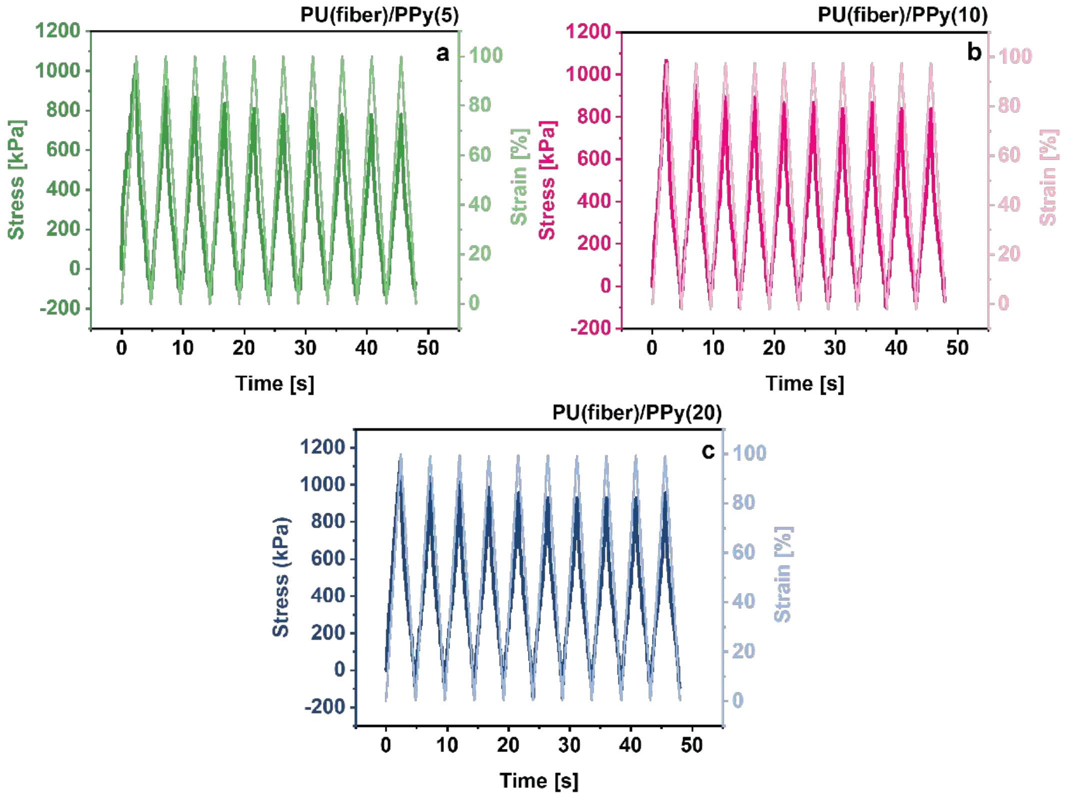


**Figure S8.** Cyclic stress response to 100% applied strain for 10 consecutive cycles of oCVD polypyrrole (PPy) coated PU fiber-mats (fiber) for **a.** 5; **b.** 10; and **c.** 20 minutes.

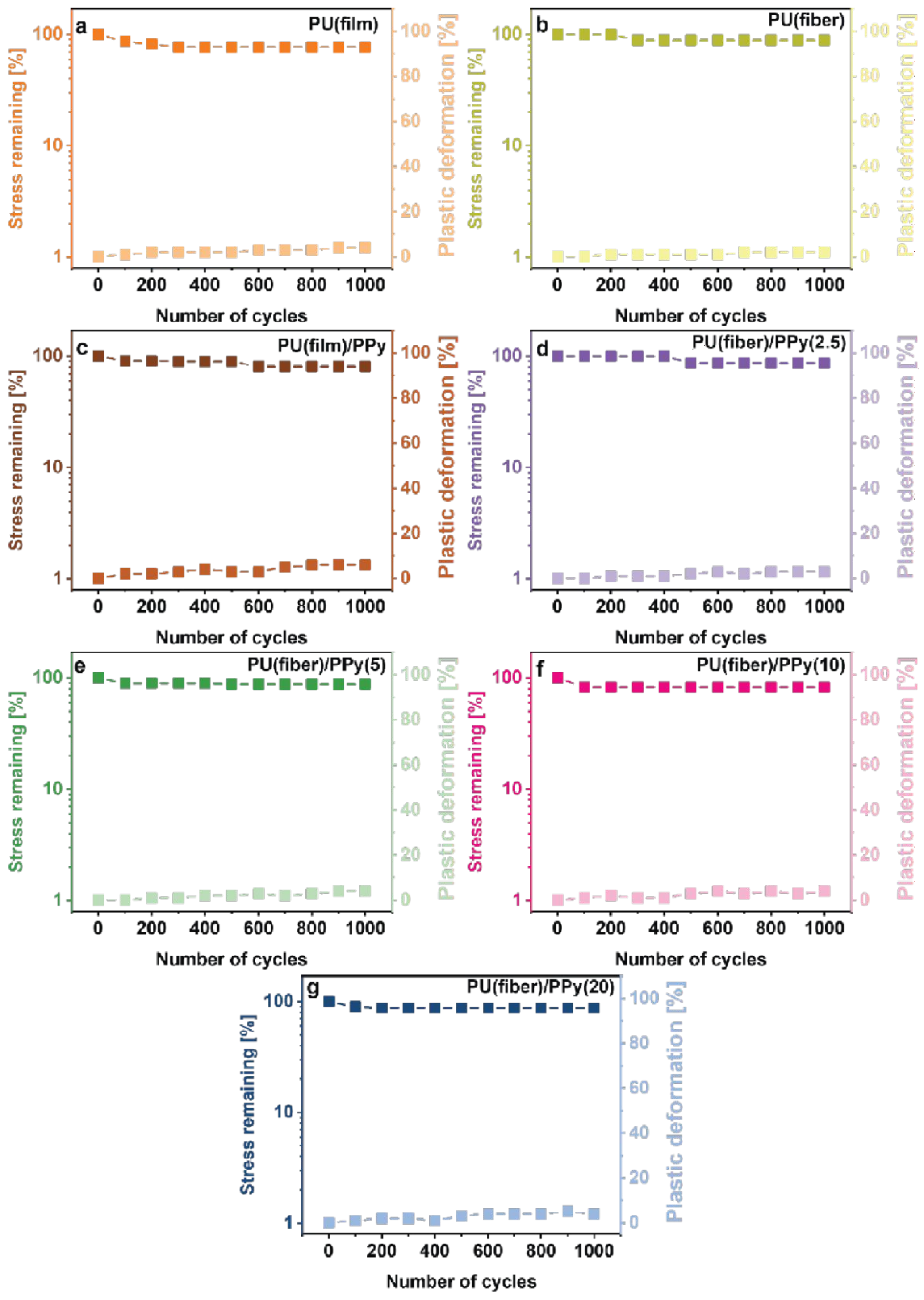


**Figure S9.** Change of maximum stress and plastic deformation over 1000 consecutive cycles of 25% applied strain showing fatigue resistance of oCVD polypyrrole (PPy) coated – **c.** PU solid

films (film); and PU fiber-mats (fiber) for **d.** 2.5; **e.** 5; **f.** 10; and **g.** 20 minutes, respectively. The response of the pristine counterparts **a.** PU (film); and **b.** PU (fiber) has been used for comparison.

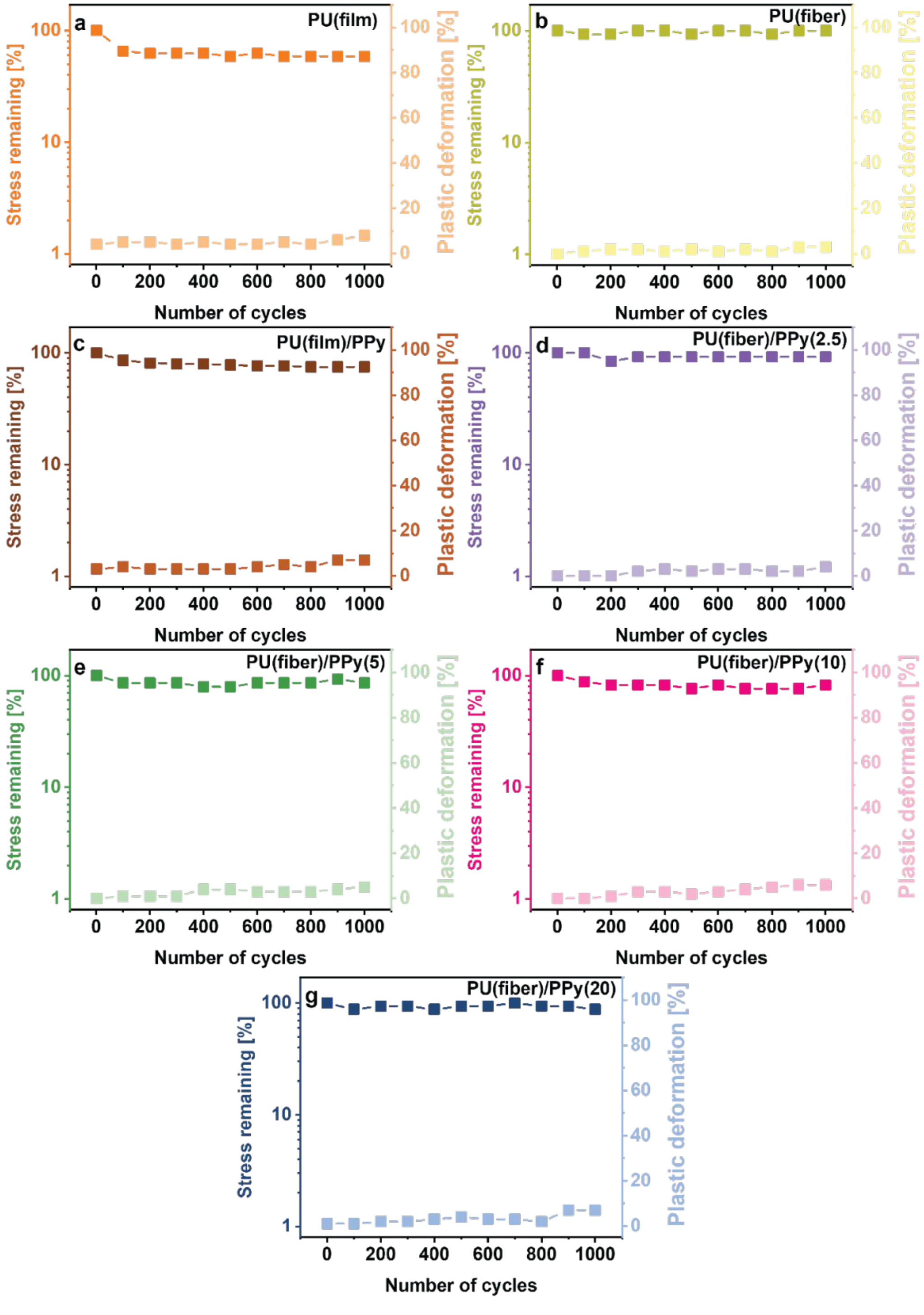


**Figure S10.** Change of maximum stress and plastic deformation over 1000 consecutive cycles of 50% applied strain showing fatigue resistance of oCVD polypyrrole (PPy) coated – **c.** PU solid

films (film); and PU fiber-mats (fiber) for **d.** 2.5; **e.** 5; **f.** 10; and **g.** 20 minutes, respectively. The response of the pristine counterparts **a.** PU (film); and **b.** PU (fiber) has been used for comparison.

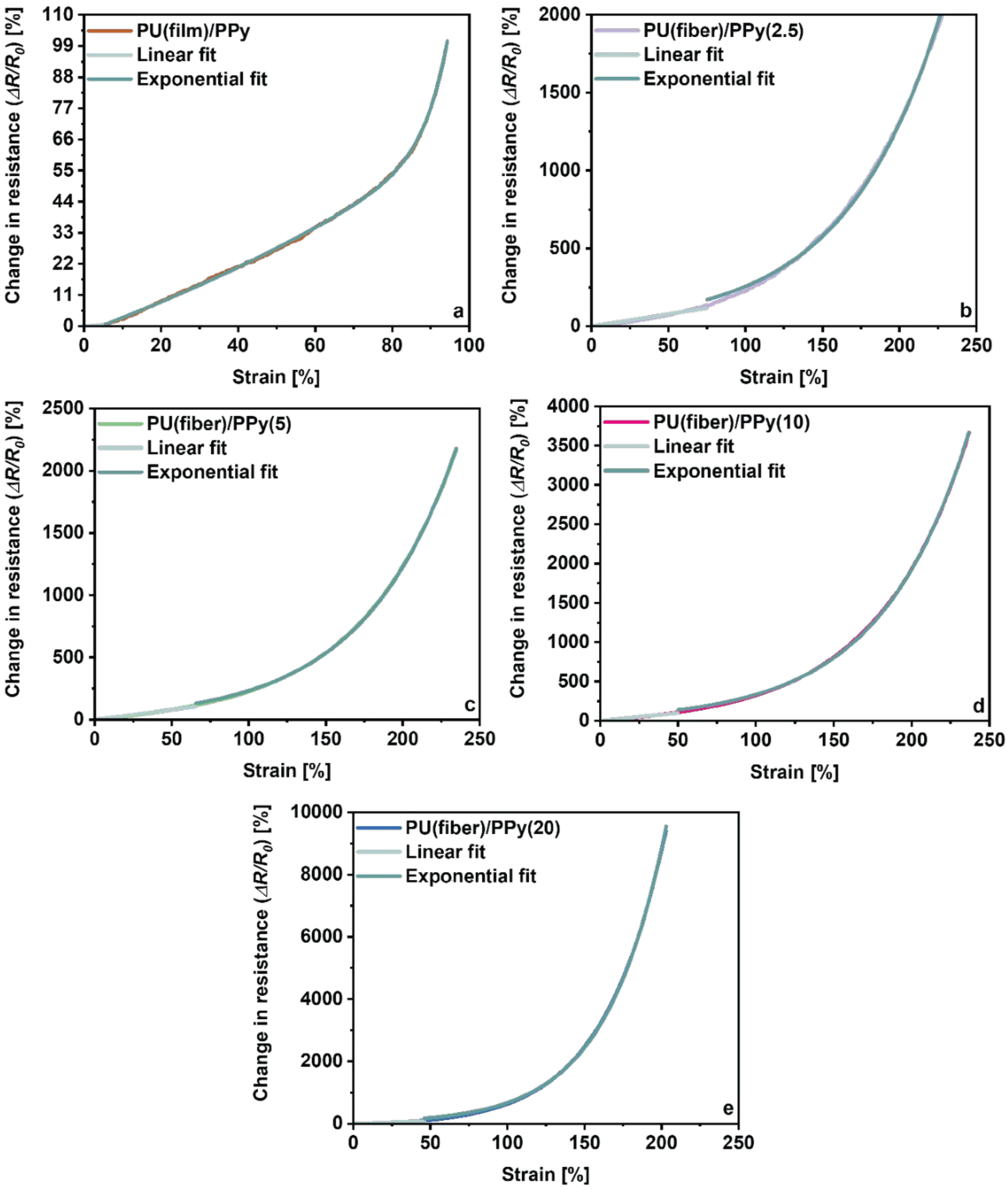


**Figure S11.** Numerical curve fitting of the mechanoelectrical response of oCVD polypyrrole (PPy) coated **a.** PU solid films (film); and electrospun fiber-mats (fiber) for different coating durations

of **b.** 2.5 min; **c.** 5 min; **d.** 10 min; and **e.** 20 min exhibiting a linear relationship for lower strains and an exponential behavior at higher strain regimes.

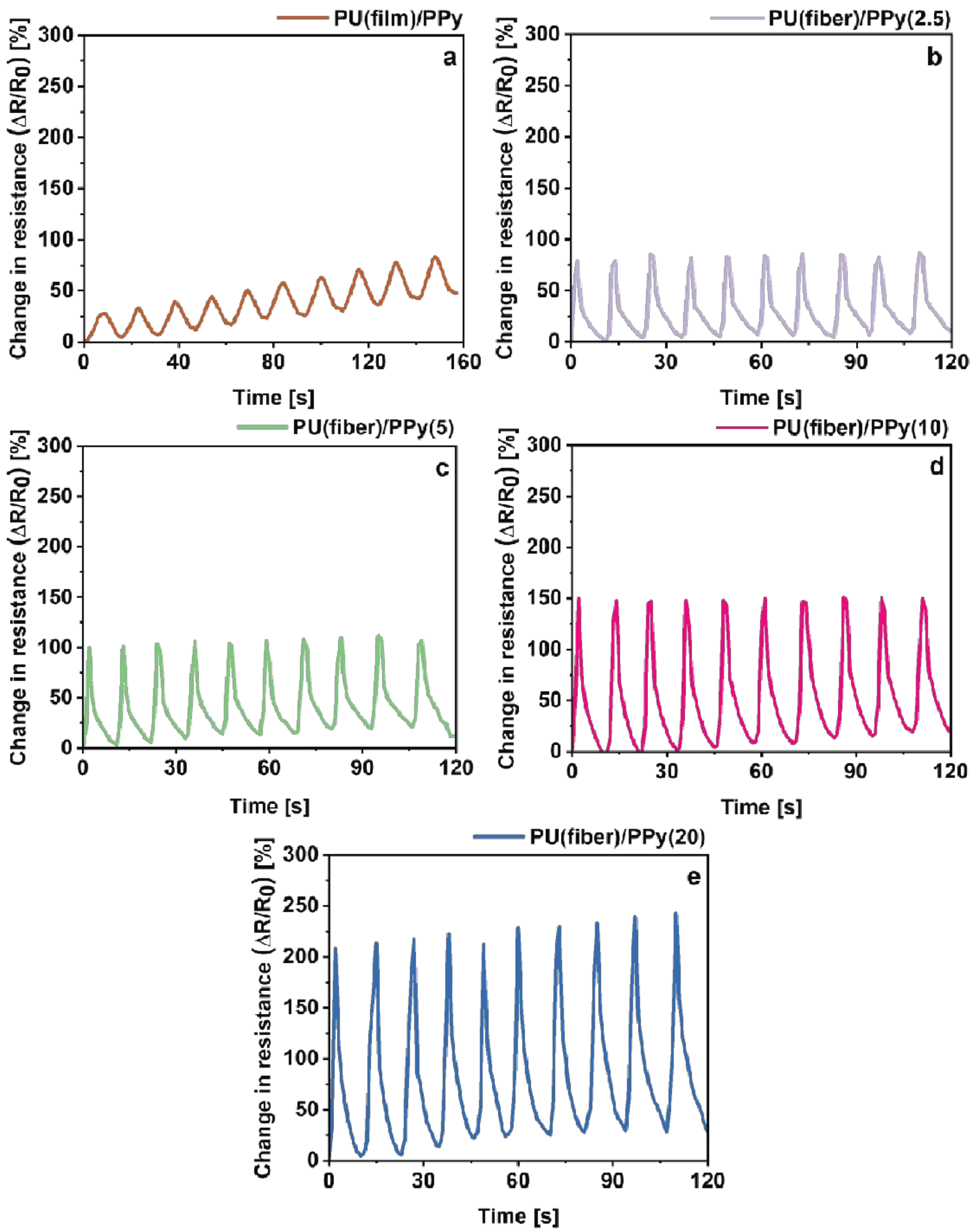


**Figure S12.** Cyclic piezoresisitive response to 50% applied strain for 10 consecutive cycles for oCVD polypyrrole (PPy) coated **a.** PU solid film (film); and PU fiber-mats (fiber) for **b.** 2.5, **c.** 5, **d.** 10; **e.** 20 minutes, respectively.

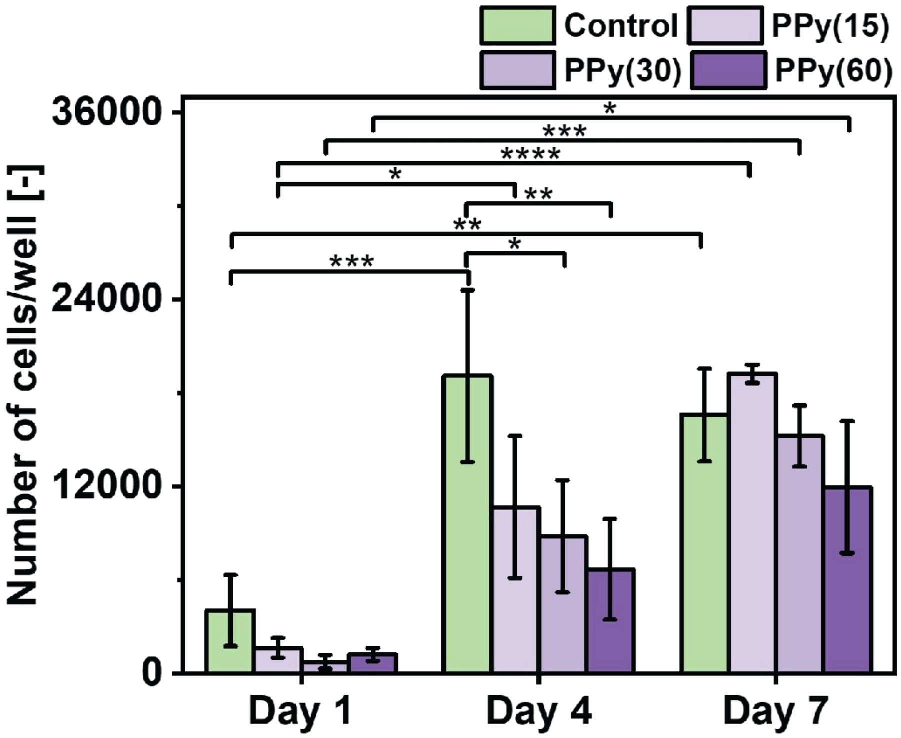


**Figure S13.** Cell number per well based on calculations from fluorescence microscopy images of HDF cells stained with PI and FDA. The names PPy(15), PPy(30), and PPY(60) correspond to the oCVD PPy-coated surface for 15, 30, and 60 minutes, respectively. Control refers to the cells cultured on an uncoated surface.

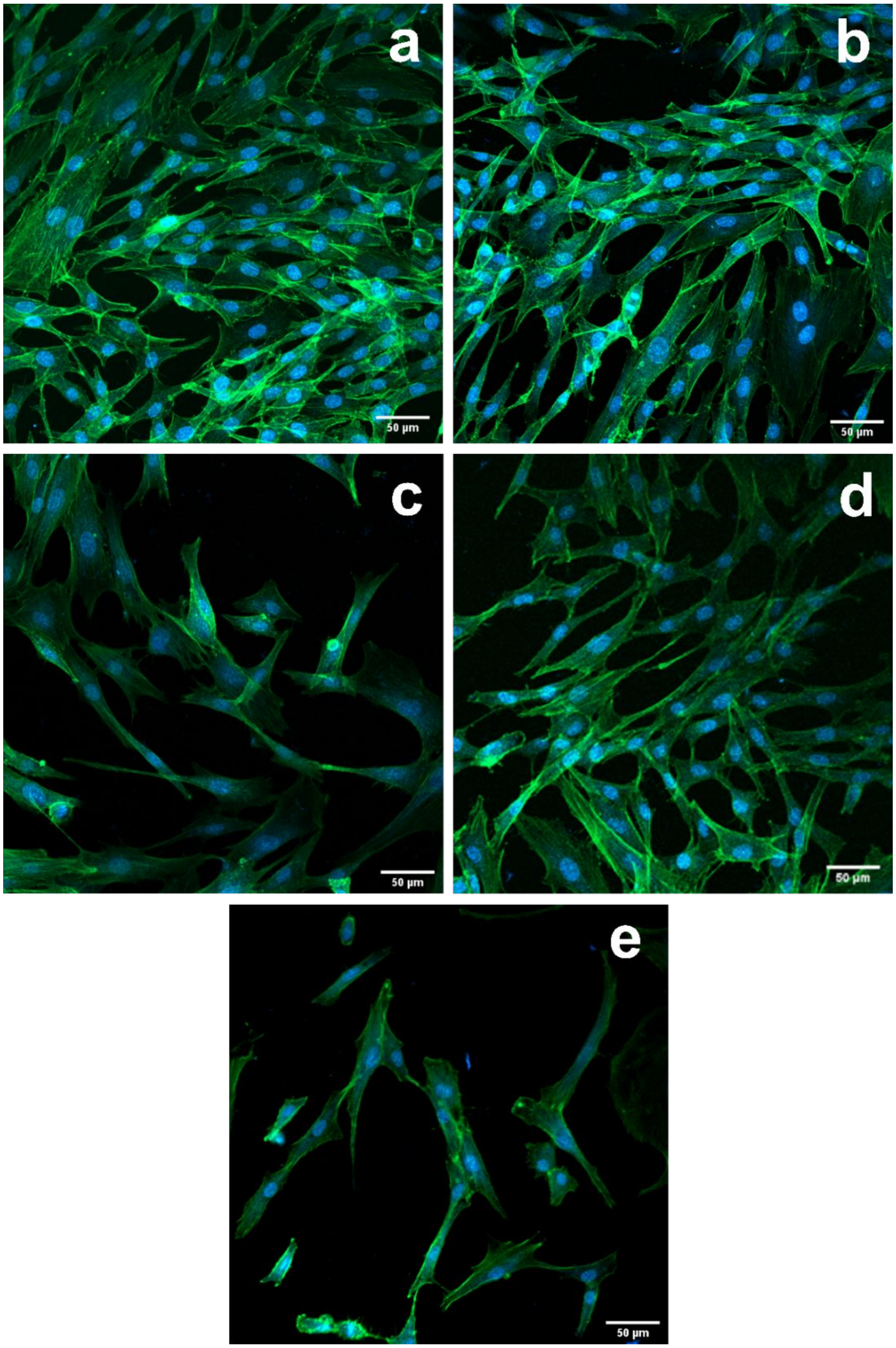


**Figure S14.** Fluorescence images of HDF cells stained with Hoechst nuclei staining (blue) and phalloidin actin staining (green). HDF cultured for 4 days on **a.** uncoated surface; and oCVD PPy-

coated surface for **b.** 15; **c.** 30; and **d,e.** 60 minutes (different spots on sample). The scale bar for all images is 50 μm.

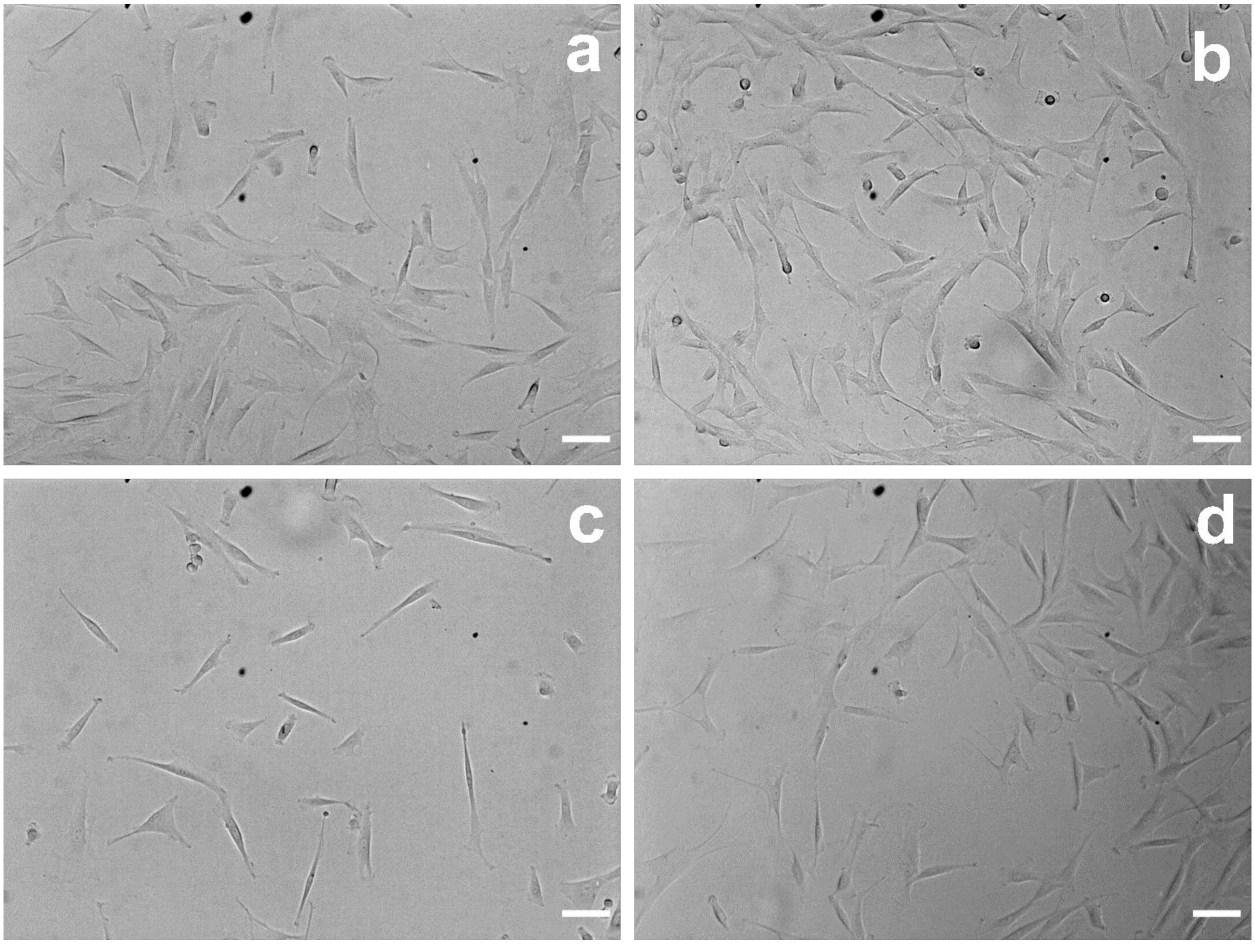


**Figure S15**. Brightfield images of HDF cells cultured for 4 days on **a.** uncoated surface; and oCVD PPy coated surface for **b.** 15; **c.** 30; and **d.** 60 minutes. The scale bar for all images is 100 μm.

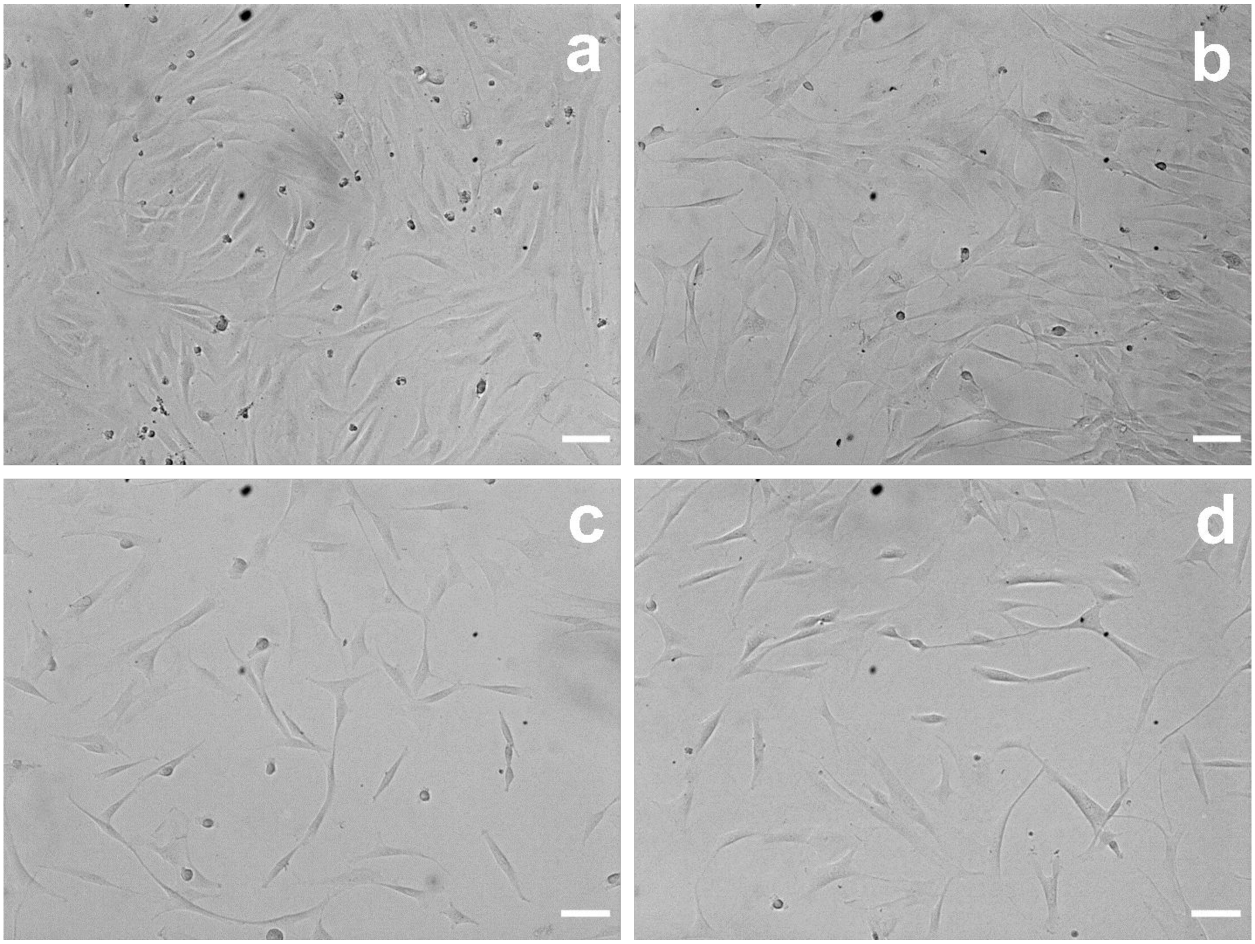


**Figure S16.** Brightfield images of HDF cells cultured for 7 days on **a.** uncoated surface; and oCVD PPy-coated surface for **b.** 15; **c.** 30; and **d.** 60 minutes. The scale bar for all images is 100 μm.
